# Tibetan medicine Bang Jian: a comprehensive review on botanical characterization, traditional use, phytochemistry, and pharmacology

**DOI:** 10.3389/fphar.2023.1295789

**Published:** 2023-12-14

**Authors:** Yuan Li, Jie Zhang, Jin-ya Fan, Shi-hong Zhong, Rui Gu

**Affiliations:** ^1^ School of Pharmacy, Chengdu University of Traditional Chinese Medicine, Chengdu, China; ^2^ College of Pharmacy, Southwest Minzu University, Chengdu, China; ^3^ State Key Laboratory of Southwestern Chinese Medicine Resources, Chengdu University of Traditional Chinese Medicine, Chengdu, China; ^4^ School of Ethnic Medicine, Chengdu University of Traditional Chinese Medicine, Chengdu, China

**Keywords:** Bang Jian, botanical characterization, traditional use, phytochemistry, pharmacological activities

## Abstract

Tibetan medicine Bang Jian refers to a range of botanical drugs within the *Gentiana* genus. It serves as a prominent traditional Tibetan botanical drug primarily found in the ethnic minority regions of the Qinghai-Tibet Plateau in China. Traditionally, the dried flowers of Bang Jian, known as “Longdanhua” have been employed in Tibetan medicine to address detoxification, pharyngeal relief, acute and chronic bronchitis, bronchiectasis, lung infections, pulmonary fibrosis, and throat disorders. Surprisingly, there has been no comprehensive review published to date on Tibetan medicine Bang Jian. This passage systematically presents and critically assesses recent advancements in botanical characterization, traditional applications, phytochemistry, pharmacology, and clinical uses of Bang Jian, aiming to provide a scientific foundation for its reasonable use and further exploration. To date, researchers have isolated and identified 92 structurally diverse compounds, with a predominant presence of iridoids, flavonoids, xanthones, and triterpenoids. The crude extracts and metabolites derived from Bang Jian have been found to exhibit a wide range of pharmacological effects, encompassing anti-inflammatory, anti-tumor, anti-bacterial, antiviral, antioxidant, hepatoprotective properties, and protect the respiratory system. Nevertheless, detailed data on the biological effects, metabolic activities, and mechanistic research concerning active monomer metabolites remain insufficient. Consequently, there is a pressing need for comprehensive and in-depth research to guide rational clinical drug usage and evaluate the medicinal attributes of Bang Jian.

## Graphical Abstract

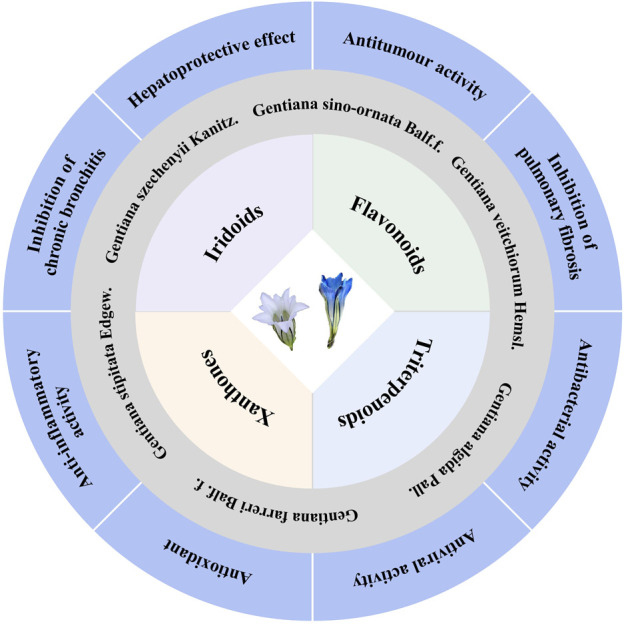

## 1 Introduction

Approximately 400 species fall within the genus *Gentiana*, widely dispersed across Southeast Asia and Europe (Zhang H. T. et al., 2009). Predominantly, they thrive at high altitudes in regions like Gansu, Sichuan, Yunnan, and other parts of China (Committee for the Flora of China, 2001). The roots and rhizomes of Gentian plants are frequently used in Chinese medicine to treat jaundice and eczema and are thought to have the effect of purging liver and gallbladder fire. Interestingly, while Tibetan medicine often uses the flowers or whole grasses of Gentian plants to treat cough and remove heat from the lungs (Bai et al., 2016). It involves a variety of Gentian plants, which are collectively referred to as “Bang Jian” in Tibetan medicine literature.

Bang Jian, which translates to “beautification of the grassland,” originates from the blossoms of various gentian plants within the *Gentiana* genus of the Gentianaceae family. Bang Jian primarily thrives in Tibet, Gansu, Qinghai, Sichuan, and Yunnan provinces (Committee for the Flora of China, 2001). Tibetan medicine literature often adopts the form of “classification.” The categorization of Bang Jian often hinges on the flower’s color, with the Jingzhu Materia Medica identifying three categories: white, blue, and black (Dimaer, 1986). However, the Bang Jian category in Tibetan botanical drugs comprises several species with intricate root origins. Prominent species include the white-flowered gentians *G. szechenyii* Kanitz. (*Gentiana szechenyii*), *G. algida* Pall. (*Gentiana algida*), and *G. stipitata* Edgew. (*Gentiana stipitata*), along with the blue-flowered gentians *G. veitchiorum* Hemsl. (*Gentiana veitchiorum*), *G. sino-ornata* Balf.f. (*Gentiana sino-ornata*), and *G. lawrencei* Burkill. (*Gentiana lawrencei*). A wide array of metabolites has been isolated from Bang Jian, encompassing iridoids, flavonoids, triterpenoids, xanthones, and organic acids. Notably, iridoids stand out as the most active metabolites, serving as markers to gauge Bang Jian’s quality. Beyond its chemical complexity, Bang Jian has demonstrated a broad spectrum of biological functions. Modern pharmacological investigations have revealed its anti-inflammatory, anti-tumor, anti-bacterial, anti-viral, hepatoprotective, and respiratory protection. Clinically, it effectively addresses bronchitis, lung infections, bronchiectasis, pulmonary fibrosis and throat diseases (Liu et al., 2006; Zhang et al., 2014; Zhao et al., 2018).

These distinctive conditions contribute to its unique potency and efficacy. Typically found at high altitudes in alpine meadows or scrubland (Committee of State Administration of Traditional Chinese Medicine for Chinese Materia Medica, 2002), some Bang Jian species face resource depletion due to overharvesting, endangering their survival. *G. szechenyii*, for instance, is classified as a first-class endangered Tibetan medicine (Zhong et al., 2014). Therefore, it is crucial to raise awareness of the necessity for conserving this species by strengthening Bang Jian’s protection measures and maximizing its utilization efficiency. With the ongoing progress and development in science, researchers should also actively conduct experiments to explore artificial cultivation methods suitable for Bang Jian, thus promoting its survival and sustainable use. Besides, Modern research has demonstrated the intricate chemical composition of traditional Chinese medicines, and there has been continuous advancement in analytical techniques to glean further insights into these medicinal substances. While existing literature has reported on the biological activity and chemical makeup of Bang Jian, recent advancements in technology have enabled the discovery of new compounds and facilitated more in-depth exploration of its pharmacological properties and clinical applications.

At present, some scholars have summarized the ethnic medicinal uses, phytochemistry and pharmacological activities of Gentianaceae in Tibetan Medicine. They mainly include Bang Jian, Ji Jie, Di Da, and Gang Ga Qiong. The inter-species differences are compared mainly for different species of Gentianaceae Tibetan medicines. However, comparisons of different origins of the same species are lacking. In addition, the pharmacological activities of Bang Jian are mostly concentrated on the prevention of upper respiratory tract infections (Chi et al., 2021). Other pharmacological activities of Bang Jian were not involved. In conclusion, the phytochemistry and other pharmacological activities of different origins plants in Bang Jian botanical drugs need to be further summarized.

Besides, as Bang Jian is the most species of Gentianaceae in Tibetan medicine, the market circulation is complicated. And there are also mixed uses in hospitals. What’s more, Bang Jian is a multi-original species. Its specific botanical characteristics, phytochemistry and pharmacological activity are different between each of the different species. This may affect the basis for its use in clinical practice. Therefore, it is necessary to carry out a more detailed and specific review of the Bang Jian of Tibetan botanical drugs. To the best of our knowledge, a comprehensive review of Tibetan medicine Bang Jian is currently absent. Consequently, this review seeks to consolidate more comprehensive knowledge from existing literature concerning Bang Jian’s traditional usage, phytochemistry, pharmacological effects, and clinical research. Its aim is to establish a theoretical foundation for the reasoned advancement and utilization of Bang Jian, including the exploration of novel dosage forms.

## 2 Methods

The relevant information on Bang Jian was collected from PubMed, Web of Science, ScienceDirect, Google Scholar, Springer, Elsevier, CNKI, Baidu Academic, JSTOR and CBM database. Other literature sources include the Chinese Pharmacopoeia and Tibetan medicine classical books. Firstly, the keywords used included *Gentiana* (Tourn.) L, *G. szechenyii* Kanitz., *G. algida* Pall., *G. stipitata* Edgew., *G. veitchiorum* Hemsl., *G. sino-ornata* Balf.f., *G. lawrencei* Burkill., Longdanhua, Bang Jian (Longdanhua of Tibetan name), without any other restrictions, a total of 242 related documents were searched. Subsequently, on this basis, the search was repeated again with botanical characterization, traditional uses, phytochemistry, and pharmacological activity as secondary keywords, respectively. Finally, 91 representative relevant literatures were obtained. All of these are included in this review.

## 3 Botanical characterization

The Tibetan medicine Bang Jian is a collective name for part of the medicinal plants of the genus *Gentiana*. It is in the genus *Gentiana* mainly includes *Gentiana* Sect. *Frigida* (*G. algida*, *G. algida* var. *purdomii* (C.Marquand) T.N.Ho, *Gentiana atuntsiensis* W.W.Sm., etc.), Sect. Monopodiae Ser. Ornatae (*G. veitchiorum*, *G. sino-ornata*, *G. lawrencei*, etc.), Sect. Monopodiae Ser. Confertifoliae (*G. szechenyii*, *Gentiana stipitate*, *Gentiana confertifolia* C.Marquand, etc.) (Committee for the Flora of China, 2001). It has a long history of medicinal use in Tibetan medical treatment and is representative of the commonly used bulk of Tibetan medicinal botanical drugs, which are contained in many Tibetan medical classics and Materia medica.

Tibetan medicine literature often according to the “classification type” form to record the list: such as the “Jing Zhu Materia Medica” records, the list according to color is divided into three kinds of white, blue, and black flowers, called “Bang Jian Babao,” “Bang Jian Wanbao” and “Bang Jian Nabao,” and the “Four Medical Tantra Blue Sapphire” is divided into three types of white, blue, and miscellaneous flowers, miscellaneous flowers called Bang Jian Chawu (Disi, 2012). Although there are flower color classifications in the literature, Tibetan medicine in the actual recognition of the field and the use of medicinal botanical drugs often dilutes the blue flower and black flower distinction. In our group’s preliminary investigation of the Bang Jian of three types of inter-list varieties of wild resources, Tibetan medicine market, and other investigations found that a total of 22 species, 4 varietas, including white-flowered gentian collated 8 species, 1 varietas, blue-flowered gentian collated 11 species, 2 varietas, black-flowered gentian 4 species, miscellaneous flowers gentian 5 species, 1 varietas (Zhong et al., 2014; Fu et al., 2018).

**FIGURE 1:**
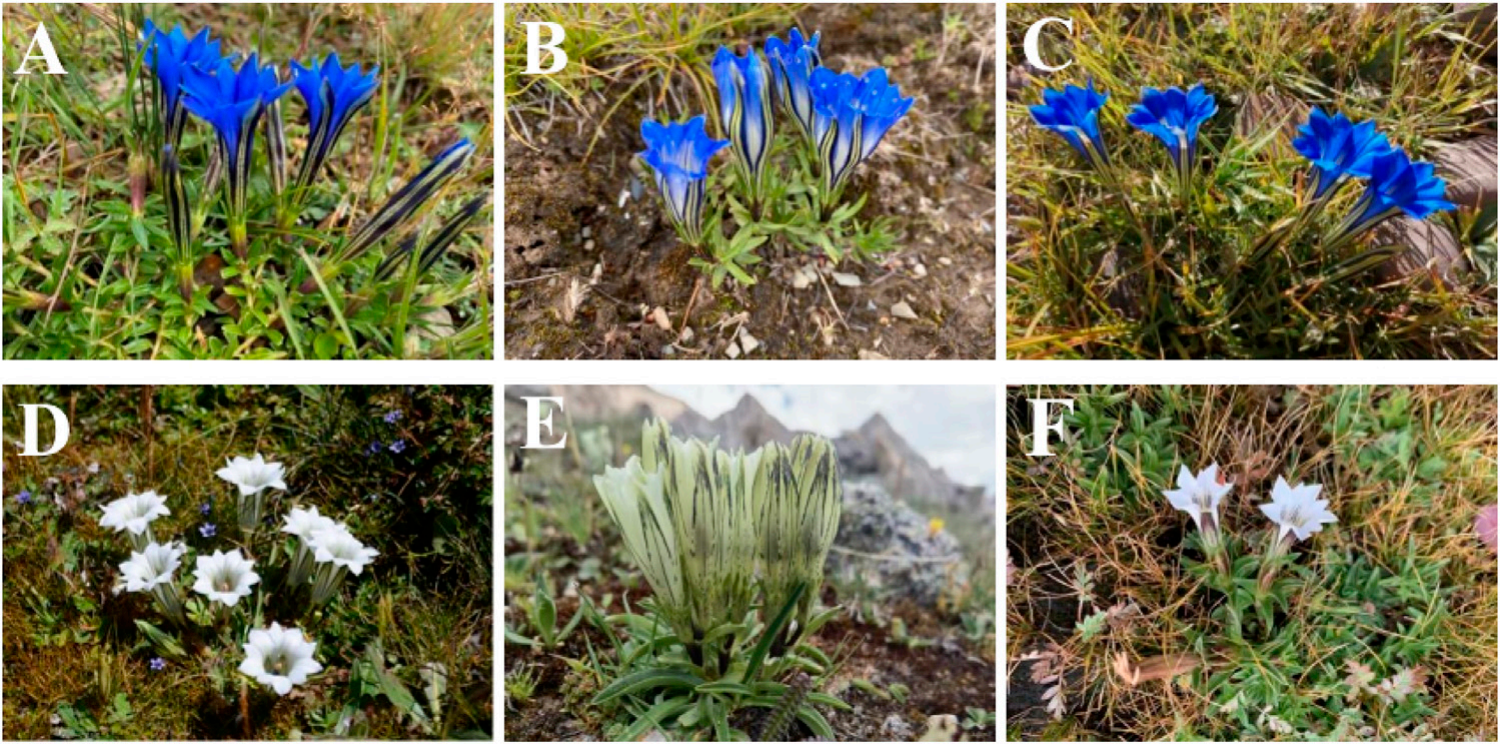
The plant pictures of mainstream varieties of Bang Jian. **(A)** *Gentiana szechenyii Kanitz.*, **(B)** *Gentiana algida* Pall., **(C)** *Gentiana stipitata* Edgew., **(D)** *Gentiana veitchiorum* Hemsl., **(E)** *Gentiana sino-ornata* Balf.f., **(F)** *Gentiana lawrencei* Burkill.

However, due to the complexity of the gentian plant, the three types of Tibetan medicine recorded in various Materia medica Bang Jian identification description are not clear, the source is complex, the classification is confusing, and there is a certain degree of subjectivity. The main species are *G*. *szechenyii*, *Gentiana purdomii*, *G. algida*, etc (Fu et al., 2018). In brief, several scholars on its varieties of evidence, the results of several scholars’ examination of its species, as well as literature, specimen records, herbal market and local Tibetan hospital visits and investigations, found that *G. szechenyii*, *G. algida,* and *G. stipitate* are the mainstream varieties of white-flowered gentian, and that the mainstream varieties of blue-flowered gentian are *G. veitchiorum*, *G. sino-ornata*, and *G. lawrencei* (Figure 1) (Zhong et al., 2014; Tang et al., 2020). Some of the morphological characteristics are similar although Bang Jian is known as a taxonomically and phylogenetically challenging taxon.

According to “Du Mu Materia Medica” Born in a rocky meadow, with small leaves and numerous flowers (Xi, 2016). The “Four Medical Tantra Blue Sapphire” records that the leaves are small and green, elliptical in shape, and the stems are thin (Disi, 2012). Based on “Jing Zhu Materia Medica,” white-flowered gentian grows on grassy slopes. The leaves are small, and the flowers are prosperous. The white-flowered gentian taste bitter which can cure febrile communicable disease, the blue-flowered gentian grows in very humid swampy grasslands in early autumn, with small leaves and light blue flowers, which are obvious, the black-flowered gentian grows in alpine meadows in mid-autumn, with a cyan surface on flowers, slightly larger than blue-flowered gentian. In addition, there is a mythological story about the flowering time of the three types of Tibetan medicine, white, blue, and black flowers of Bang Jian. It is believed that there are “three sisters of the fairy” in the autumn when all the drugs withered, the flowers withered, then turned into the list of three types of gentian, as a graphic description of the flowering time of the list of Tibetan medicine (Dimaer, 1986).

Based on recent morphological studies of medicinal botanical drugs in “Bang Jian,” it has been found that, specifically, blue-flowered gentian is a perennial botanical drug with medium sized flowers and a few larger ones. The sepals are short, about one-third to one-half the length of the corolla, and the lobes are often shorter than the sepal tube, rarely as long as it. Most of the flower branches have flowers. Corolla funnel-shaped, sparsely inverted conical, 4–6 cm long. Their difference lies in the fact that the leaves in the middle and lower parts of the stem of the *G. veitchiorum* are wider, oval, or ovate lanceolate in shape, with a sharp apex but no small tip (Dimaer, 1986; Zhang, 2016). The stem leaves in the middle and lower parts of *G*. *lawrencei* and *G. sino-ornata* are narrow, linear-rectangular, or lanceolate, and the apex is acute. The corolla is light blue, with a yellowish white bottom and no spots. The difference between the two lies in the fact that there are no undeveloped twigs in the axils of the leaves of the *G*. *lawrencei*. And the leaves are often sparse, with finely linear in shape. There are very undeveloped branchlets in the leaf axils of *G. sino-ornata.* The leaves densely packed, linear lanceolate. *G. stipitate* and *G. szechenyii* have short stems. The leaves are densely packed in a lotus shape. The main root is thick, conical, or cylindrical. The lotus shaped leaf clusters of sterile stems are wider. Leaves and calyx lobes are broad with distinct cartilaginous edges (Wu and Wang, 2017). Most of the flower branches are clustered and scattered, with a length of 7–10 cm; There are many pairs of stem leaves, with sparse lower parts and dense upper parts. The calyx lobes are oblanceolate, and the base shrinks. The corolla of *G. stipitate* is light blue gray. The branches are few and straight extending, 2–3 cm long. A few flower branches, straight extending, 2–3 cm long. The stem leaves are few and dense. The base of the calyx lobes is not narrowed. The corolla has deep blue gray wide stripes and spots, with white inside. And the calyx lobes of *G. szechenyii* are lanceolate (Ma et al., 2015). Unlike *G. szechenyii* and *G. stipitate*, *G. algida* have rhizomes or creeping stems, with well-developed rosette shaped leaf clusters (Ma et al., 2017). The shape of stem leaves is the same as the leaves of the lotus leaf cluster. The seeds are reticulately spongy and wingless. The calyx lobes are erect and neat with 1–3 (−5) flowers, and usually have sessile or shortly pedicels. The corolla has blue spots and no stripes. Summarized information on the characteristics of Bang Jian is given in Table 1 and Supplementary Material.

**TABLE 1:** Summary of botanical characteristics and pictures of specimens of the dominant species of Bang Jian.

Classification	Species	Similarity	Differential	Herbarium pictures	Voucher no.
Blue flower	*G. veitchiorum*	① Flowers: medium, and a few larger ones	① The leaves in the middle and lower parts of the stem are wider, oval, or ovate lanceolate in shape, with a sharp apex but no small tip	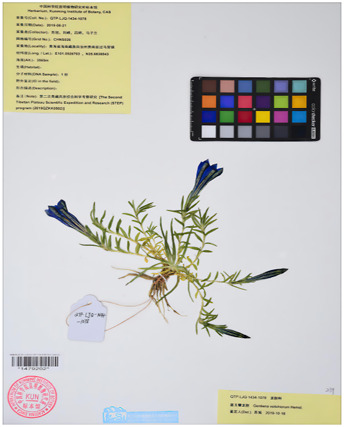	KUN1479202
		② Sepals: short, about 1/3 to 1/2 the length of the corolla, and the lobes are often shorter than the sepal tube, rarely as long as it	② Corolla: dark blue		
		③ Most of the flower branches have flowers, corolla funnel-shaped, sparsely inverted conical, 4–6 cm long			
	*G. lawrencei*	① Flowers: medium, and a few larger ones	① No undeveloped twigs at the leaf axils	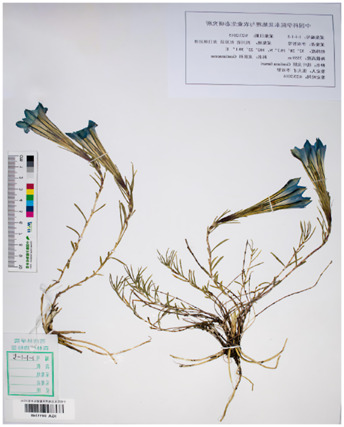	IGA0011348
		② Sepals: short, about 1/3 to 1/2 the length of the corolla, and the lobes are often shorter than the sepal tube, rarely as long as it			
		③ Most of the flower branches have flowers, corolla funnel-shaped, sparsely inverted conical, 4–6 cm long	② Leaves: sparse with finely linear in shape		
		④ The stem leaves in the middle and lower parts are narrow, linear-rectangular, or lanceolate, and the apex is acute			
		⑤ Corolla: light blue, with a yellowish white bottom and no spots			
	*G. sino-ornata*	① Flowers: medium, and a few larger ones	① Undeveloped branchlets at the leaf axils	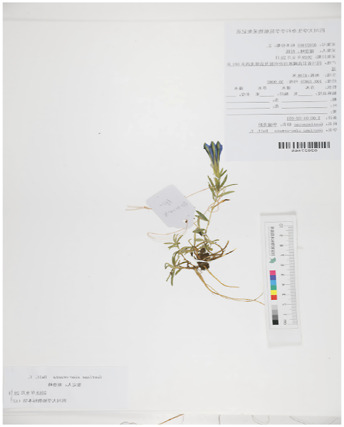	SZ02027465
		② Sepals: short, about 1/3 to 1/2 the length of the corolla, and the lobes are often shorter than the sepal tube, rarely as long as it			
		③ Most of the flower branches have flowers, corolla funnel-shaped, sparsely inverted conical, 4–6 cm long	② Leaves: crowded with linear-lanceolate in shape		
		④ The stem leaves in the middle and lower parts are narrow, linear-rectangular, or lanceolate, and the apex is acute			
		⑤ Corolla: light blue, with a yellowish white bottom and no spots			
White flower	*G. szechenyii*	① Slightly fleshy fibrous roots	① Branches: few and straight extending, 2–3 cm long	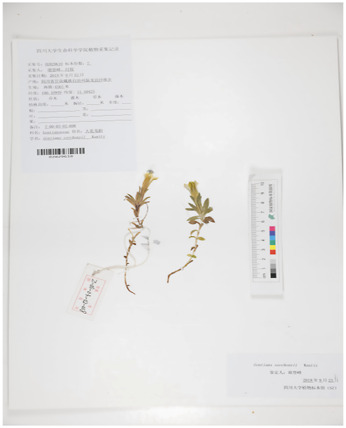	SZ02029610
		② Short stems			
		③ Leaves: densely packed in a lotus shape	② Stem leaves: few and dense		
		④ Main root: thick, conical, or cylindrical	③ Calyx lobes: lanceolate, and not contracted at base		
		⑤ The lotus shaped leaf clusters of sterile stems are wider. Leaves and calyx lobes are broad with distinct cartilaginous edges	④ Corolla: deep blue gray wide stripes and spots, with white inside		
		⑥ Seeds: dark brown with light honeycomb reticulation on the surface			
	*G. stipitate*	① Slightly fleshy fibrous roots	① A lot of flower branches, spreading out, with a length of 7–10 cm	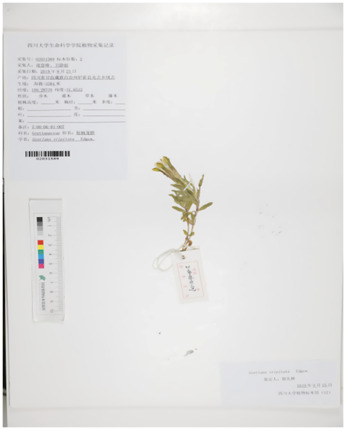	SZ02031589
		② Short stems			
		③ Leaves: densely packed in a lotus shape	② Many pairs of stem leaves, with sparse lower parts and dense upper parts		
		④ Main root: thick, conical, or cylindrical	③ Calyx lobes: oblanceolate, and the base shrinks		
		⑤ Lotus shaped leaf clusters of sterile stems are wider. Leaves and calyx lobes are broad with distinct cartilaginous edges	④ Corolla: light blue gray		
		⑥ Seeds: dark brown with light honeycomb reticulation on the surface			
	*G. algida*	① Slightly fleshy fibrous roots	① Rhizomes or creeping stems, with well-developed rosette shaped leaf clusters	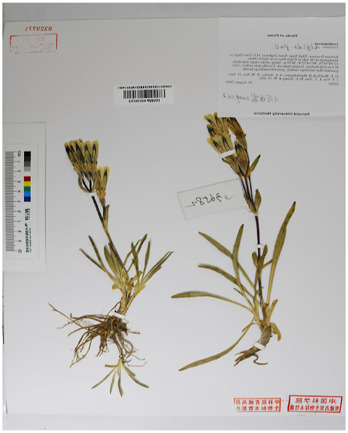	HNWP00026752
			② The shape of stem leaves is the same as the leaves of the lotus leaf cluster		
			③ The number of flowers are usually 1–3, sparsely up to 5, terminal		
			④ Calyx lobes: erect and neat		
			⑤ Corolla: blue spots and no stripes		
			⑥ Seeds: yellowish brown with spongy reticulation on the surface		

All species mainly grow on grassy slopes and forest margins at an altitude of 2,000–5,000 m in Gansu, Qinghai, Sichuan, Tibet, and Yunnan of China, while foreign distribution is mainly in India, northeastern Myanmar, the Soviet Union, Japan, and Nepal (Committee for the Flora of China, 2001).

As the Tibetan medicine Bang Jian is a multi-basic original plant, and there are more confusing products, the traditional identification method mainly based on morphological characteristics has its limitations. It is well known that DNA barcode technology has the advantages of not relying on the morphology of botanical drugs, excluding the subjective factors of the identifier, good reproducibility of the identification results, good versatility of the method, and easy to promote and standardize, etc (Kress, 2017; Fernandes et al., 2021; Yu et al., 2021). ITS sequence is one of the most commonly used molecular markers, which is widely used in the construction of phylogenetic tree and the analysis of population genetic structure. Our group constructed a phylogenetic tree by MEGA6.06 by combining the ITS sequences of the mainstream varieties of white-flowering gentian with the ITS2 sequences of the easily mixed varieties of white-flowering gentian retrieved from the GenBank database. The results showed that it was possible to separate the *G. szechenyii* from the *G. algida*, and that the ITS2 sequence could be used as a DNA barcode of *G. szechenyii* for the identification of *G. szechenyii* and its mixed forgeries (Zhang, 2016). However, it was also found that the ITS sequences of *G. veitchiorum* and *G*. *lawrencei* were highly heterozygous, and it was difficult to distinguish these two species by molecular fragment ITS, and further monoclonal sequencing was needed (Sun and Wang, 2019; Fu et al., 2020). It provides a reference for the barcode study and differentiation analysis of Tibetan medicine of Bang Jian.

## 4 Traditional uses

In China, as one of the traditional Tibetan medicines, Bang Jian has been recorded in many monographs on Tibetan medicines. According to textual research, as early as the 14th century AD, it was recorded in the Materia Medica Library: “It is believed that it is named according to its growing environment, such as Wawei and Yangou grass.” “Bang” refers to alpine meadow, and “Jian” means decoration. Ethnomedical applications of Bang Jian trace back to the middle of the eighth century B.C. Its medical values have been firstly recoded in the “Yue Wang Yao Zhen” that “Bang Jian” is used in the formula for lung diseases (Mao, 2012). For example, the medical book “Jing Zhu Materia Medica” records that the Bang Jian has a little astringent in taste, cool in nature, clears lung heat, treats poisonous diseases, cures all feverish diseases, detoxifies, and facilitates the throat. It has been recorded that blue flowers produce the same effect as white-flowered gentian but are cool in nature. The black one also has the effect of treating black scars and acne rashes, usually taken as a single flavor in water or powdered into a compound (Dimaer, 1986). In addition, Bang Jian was described detailed in the “Four Medical Tantra” reads: “Bang Jian ga bao (white flower) can cure laryngitis and detoxify heat” (Yutuo, 2005). It can be seen that “Bang Jian” has the exact effect in a very early time, mainly used for respiratory diseases. In Tibetan medicine, blue flowers are used to treat red eye headache, pharyngitis, damp heat, jaundice, and other symptoms. After investigation, the blue flower, Tibetan medicine called the *G. veitchiorum* combined with the investigation of clinical drug use, the varieties used by Tibetan hospitals and Tibetan medicine factories as *G. veitchiorum* are mainly *Gentian*, which are used as whole botanical drug.

It is also recorded in the annals of medicine of other ethnic minorities. The difference is that in other ethnic minorities, the medicinal parts are mainly concentrated on the whole botanical drug and the root (Table 2). It is recorded in “Kazak Pharmacy” that Alpine gentian *Gentiana. triflora* Pall., whole botanical drug into medicine, bitter, cold, with the function of clearing heat and detoxification, used for acute and chronic hepatitis, jaundice, cholecystitis, cystitis, hypertension, dizziness, tinnitus, and other diseases (BaHaErGuLi, 2009). “Uighur pharmacopoeia” contains gentian medicinal materials, also known as Jinti Yana, for *Gentiana scabra* Bunge., striped leaf *Gentiana manshurica* Kitag., three-flowered gentian *Gentiana triflora* Pall., strong *Gentiana crassa subsp. rigescens* (Franch. ex Hemsl.) Halda. are all rooted in medicine, with clear heat and dampness, diarrhea liver and eyes, mainly treating humid heat jaundice, scrotal itching, skin sores, eye redness and pain, high fever caused by liver fire, hand and foot spasm, seizures, and other symptoms. The roots and rhizomes of *Gentiana lutea* L. and the whole botanical drug of *G. algida* are also used for medicinal purposes (Liu and ShaWuTi, 1986). The Shangri-La Ethnic Medicine Guide contains: “*Gentiana aristata* Maxim.’s whole botanical drug is used as a Tibetan medicine” complete cloth, which has clear heat, dispel dampness, relieve cough, and mainly treat sore throat, cold fever, eczema and itching, lung fever, cough, bronchitis, and other symptoms. The root of *G. sino-ornata* Balf.f. cures nameless swelling and poison, huaqing heat and detoxification, treats influenza fever, lung fever, cough, and other symptoms (Liu and Zheng, 2008). In addition, one of the mainstream species of Bang Jian, *G. algida*, is also included in “The Haixi Mongol and Tibetan Materia Medica Resources.” And it has a history of medicinal use in the Tibetan region of Qinghai. It can be seen that Bang Jian medicinal plants have rich medicinal plant resources with distinctive national medicinal characteristics, and are widely used in Tibet, Mongolia, Wei, Kazakh, and other ethnic minorities. In a word, Bang Jian have a wide range of traditional applications. The traditional uses of common species are summarized as the treatment of detoxification, various throat disorders including lung-heat cough, bronchitis, sore throat, and various other conditions. Therefore, Bang Jian as Tibetan medicine is worthy of further in-depth excavation and investigation, to provide a guidance for modern pharmacological studies.

**TABLE 2:** Traditional applications of Bang Jian in different ethnomedicines in China.

Ethnic groups/areas	Medicinal parts	Source	Ethnomedicinal uses	References
Tibetan	flowers	*G. szechenyii*	Purification of lung heat, detoxification of toxin, relief of the throat, dipping of the actual fire in the gall bladder, treatment of seasonal fever; feverish cough, laryngitis and heat closure, poisonous diseases	Dimaer (1986)
		*G. algida*		
		*G. stipitata*		
		*G. veitchiorum*		
		*G. sino-ornata*		
		*G. lawrencei*		
Mongolian	flowers	*G. algida*	Heat clearing and toxin detoxification, cough relief and throat stimulation, sore throat, dullness, pulmonary fever, and toxic fever	Luo (1989)
		*G. veitchiorum*		
Kazak	Whole grass	*G. algida*	Heat and toxin removal, acute and chronic hepatitis, jaundice, cholecystitis, cystitis, hypertension, vertigo, and tinnitus	BaHaErGuLi (2009)
Uighur	Whole grass	*G. algida*	Clearing heat and drying dampness, diarrhea of the liver and clearing the eyes, damp-heat jaundice, redness and swelling of the eyes, constriction of the hands and feet, epilepsy etc.	Liu and ShaWuTi (1986)
Shangri-La	Roots; flowers	*G. sino-ornata*	Roots:tendon relaxation and blood revitalization to treat nameless swelling and toxicity; Flowers:Influenza, lung fever and cough, heat clearing and toxin detoxification	Liu and Zheng J. (2008)

## 5 Phytochemistry

**FIGURE 2:**
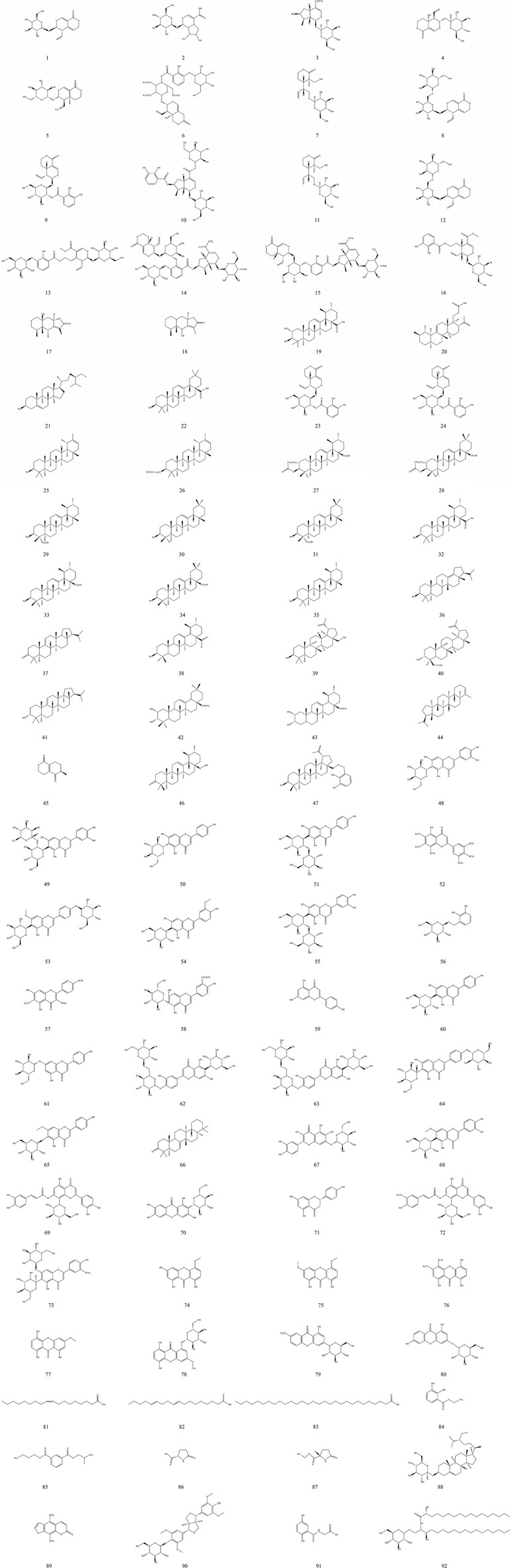
(Continued).

The medicinal effects of botanical drugs are usually mediated by a variety of bioactive metabolites. Research into the chemical composition of Tibetan medicine Bang Jian began in 1980, focusing mainly on the mainstream species. To date, more than ninety compounds have been isolated and identified by TLC, UV, IR, 1D and 2D NMR, HPLC, GC-MS, HRMS (Li, 2017; Li. et al., 2017), and other methods, mainly including iridoids (1–18), triterpenoids (19–47), flavonoids (47–73), xanthones (74–80), organic acids (81–87) and other compounds (87–92). Among them, the iridoids, flavonoids, triterpenoids, and xanthones have a strong physiological activity and are the main active metabolites in Bang Jian. A total of 92 were identified based on literature reports and chemical metabolites extracted from various medicinal parts. The names, classifications, formulation, botanical origin, and corresponding structures are shown in Table 3 and Figure 2.

**TABLE 3:** Chemical compositions of Bang Jian.

No.	Name	Classification	Source	Formula	References
1	Gentiopicroside	Iridoids	*G. algida*	C_16_H_20_O_9_	Liu et al. (2006), Yang et al., 2008; Sun (2012)
			*G. veitchiorum*		
			*G. lawrencei*		
2	Loganic acid	Iridoids	*G. veitchiorum*	C_16_H_24_O_10_	Yang et al. (2008), Bao et al. (2023)
			*G. lawrencei*		
			*G. algida*		
3	Loganin	Iridoids	*G. szechenyii*	C_17_H_26_O_10_	Zong (2015)
4	Swertiamarin	Iridoids	*G. sino-ornata*	C_16_H_22_O_10_	Sun, 2012; Ning et al. (2019)
			*G. lawrencei*		
5	Sweroside	Iridoids	*G. lawrencei*	C_16_H_22_O_9_	Sun, 2012; Yang et al. (2014b), 2015; Bao et al. (2023)
			*G. sino-ornata*		
			*G. algida*		
6	Ridoside	Iridoids	*G. algida*	C_35_H_42_O_21_	Yang et al. (2014b)
7	1-O-β-D-glucopyranosylamplexine	Iridoids	*G. algida*	C_16_H_26_O_9_	Naonobu. et al. (2015)
8	6-O-β-D-glucopyranosyl gentiopicroside	Iridoids	*G. szechenyii*	C_22_H_30_O_14_	Fu et al. (2018)
9	2’-(2,3-Dihydroxybenzoyl) sweroside	Iridoids	*G. algida*	C_23_H_26_O_12_	Fu et al. (2018)
10	Gentiournoside E	Iridoids	*G. szechenyii*	C_29_H_38_O_18_	Ma et al. (2015), Zong (2015)
			*G. stipitata*		
11	Gentiournoside D	Iridoids	*G. szechenyii*	C_23_H_28_O_13_	Ma et al. (2015), Fu et al. (2018)
			*G. stipitata*		
12	Gentiournoside A	Iridoids	*G. szechenyii*	C_40_H_52_O_22_	Zhang et al. (2022)
13	Depressine	Iridoids	*G. stipitata*	C_30_H_40_O_18_	Ma et al. (2015), Fu et al. (2018)
			*G. szechenyii*		
14	Gentizechenlioside A	Iridoids	*G. stipitata*	C_46_H_60_O_26_	Ma et al. (2015), Fu et al. (2018)
			*G. szechenyii*		
15	Szechenyin A	Iridoids	*G. stipitata*	C_40_H_50_O_21_	Ma et al. (2015), Fu et al. (2018)
			*G. szechenyii*		
16	Szechenyin B	Iridoids	*G. szechenyii*	C_24_H_30_O_13_	Zhang et al. (2022)
17	10β-hydroxy-7 (11)-eremophilen-12,8α-olide	Iridoids	*G. algida*	C_15_H_22_O_3_	Yang et al. (2013)
18	6β-hydroxy-7 (11)-eremophilen-12,8α-olide	Iridoids	*G. algida*	C_15_H_22_O_3_	Yang et al. (2013)
19	Corosolic acid	Triterpenoids	*G. algida*	C_30_H_48_O_4_	Zou et al. (2010), Yang et al., 2015; Li (2017)
			*G. veitchiorum*		
			*G. szechenyii*		
20	Roburic acid	Triterpenoids	*G. algida*	C_30_H_48_O_2_	Yang et al., 2015; Li (2017), Fu et al. (2018)
			*G. veitchiorum*		
			*G. szechenyii*		
21	β-Sitosterol	Triterpenoids	*G. algida*	C_29_H_50_O	Yang et al. (2015), Bao et al. (2023)
			*G. veitchiorum*		
22	Oleanic acid	Triterpenoids	*G. algida*	C_30_H_48_O_3_	Yang et al. (2015)
23	6’-(2,3-dihydroxybenzoyl) sweroside	Triterpenoids	*G. algida*	C_23_H_26_O_12_	Tan et al. (1997)
24	6’-(2,3-dihydroxybenzoyl) swertiamarin	Triterpenoids	*G. algida*	C_23_H_26_O_13_	Tan et al. (1997)
25	Taraxasterol	Triterpenoids	*G. algida*	C_30_H_50_O	Yang et al. (2011)
26	3β-palmitate	Triterpenoids	*G. algida*	C_46_H_80_O_2_	Yang et al. (2011)
27	3β-palmitate-28-hydroxyl-α-amyrin	Triterpenoids	*G. algida*	C_46_H_80_O_3_	Yang et al. (2011), Sun (2012)
			*G. lawrencei*		
28	3β-palmitate-28-hydroxyl-β-amyrin	Triterpenoids	*G. algida*	C_46_H_80_O_3_	Zhang et al. (2009b), Yang et al. (2011)
			*G. lawrencei*		
29	24-hydroxy-α-amyrin	Triterpenoids	*G. algida*	C_30_H_50_O_2_	Yang et al. (2011)
30	β-Amyrin	Triterpenoids	*G. algida*	C_30_H_50_O	Yang et al. (2011)
			*G. lawrencei*		
31	24-hydroxy-β-amyrin	Triterpenoids	*G. algida*	C_30_H_50_O_2_	Yang et al. (2011)
32	Ursolic Acid	Triterpenoids	*G. algida*	C_30_H_48_O_3_	Dou et al. (2020), Bao et al. (2023)
			*G. veitchiorum*		
33	28-hydroxy-α-amyrin	Triterpenoids	*G. algida*	C_30_H_50_O_2_	Zhang et al. (2009c); Yang et al. (2011); Yang et al. (2015)
			*G. lawrencei*		
34	28-hydroxy-β-amyrin	Triterpenoids	*G. algida*	C_30_H_50_O_2_	Yang et al. (2011)
			*G. lawrencei*		
35	α-Amyrin	Triterpenoids	*G. algida*	C_30_H_50_O	Yang et al. (2015)
			*G. lawrencei*		
36	Boehmerol	Triterpenoids	*G. algida*	C_29_H_48_O	Yang et al. (2015)
37	Arborinone	Triterpenoids	*G. algida*	C_30_H_48_O	Yang et al. (2015)
38	3β-Hydroxy-28-formaldehyde-12-en-ursane	Triterpenoids	*G. algida*	C_30_H_48_O_2_	Yang et al. (2015)
39	28-hydroxy-lupinol	Triterpenoids	*G. algida*	C_30_H_50_O_2_	Yang et al. (2015)
			*G. lawrencei*		
40	24-hydroxy-lupinol	Triterpenoids	*G. algida*	C_30_H_50_O_2_	Yang et al. (2012), Yang et al. (2015)
			*G. lawrencei*		
41	Arborinol	Triterpenoids	*G. algida*	C_30_H_50_O	Yang et al. (2013)
42	Methyl 3-epimaslinate	Triterpenoids	*G. algida*	C_31_H_50_O_4_	Yang et al. (2013)
43	Methy 2α,3α-dihydroxyurs-12-en-28-oate	Triterpenoids	*G. algida*	C_31_H_50_O_4_	Yang et al. (2013)
44	3-Filicene	Triterpenoids	*G. algida*	C_30_H_50_	Yang et al. (2013)
45	9-Oxo-swerimuslactone	Triterpenoids	*G. veitchiorum*	C_37_H_56_O_4_	Guo et al. (2021)
46	17-Hydroperoxide-28-norurs-12-en-3-one	Triterpenoids	*G. veitchiorum*	C_29_H_46_O_3_	Guo et al. (2021)
47	28-O-(3,4-dihydroxyl-benzyl)-lupeol	Triterpenoids	*G. veitchiorum*	C_37_H_56_O_4_	Guo et al. (2021)
48	Luteolin-6-C-glucoside	Flavonoids	*G. algida*	C_21_H_20_O_11_	Yang et al. (2008), Zou et al. (2010)
			*G. veitchiorum*		
			*G. szechenyii*		
49	Isoorientin2″-O-rhamnoside	Flavonoids	*G. algida*	C_27_H_30_O_15_	Tozhiboev et al. (2011); Zong et al. (2015), Li et al. (2016b)
			*G. veitchiorum*		
			*G. szechenyii*		
50	Isovitexin	Flavonoids	*G. veitchiorum*	C_21_H_20_O_10_	Yang et al. (2008), Zou et al. (2010)
51	Isovitexin-2″-*O*-glucopyranoside	Flavonoids	*G. szechenyii*	C_27_H_30_O_15_	Zhang (2017)
52	Gardenin A	Flavonoids	*G. algida*	C_21_H_22_O_9_	Tozhiboev et al. (2011)
53	Flavocommelin	Flavonoids	*G. algida*	C_28_H_32_O_15_	Tozhiboev et al. (2011), Li et al. (2016b)
			*G. veitchiorum*		
54	Isoscoparin	Flavonoids	*G. algida*	C_22_H_22_O_11_	Zou et al. (2010), Sun (2012), Fu et al. (2018)
			*G. veitchiorum*		
			*G. lawrencei*		
55	isoorientin-2″-*O*-glucopyranoside	Flavonoids	*G. veitchiorum*	C_27_H_30_O_16_	Sun (2012), Fu et al. (2018), Ning et al. (2019), Bao et al. (2023)
			*G. sino-ornata*		
			*G. lawrencei*		
56	2,3-dihydroxybenzyl alcohol β-glucopyranoside	Flavonoids	*G. veitchiorum*	C_13_H_18_O_8_	Bao et al. (2023)
57	3,6,4′-trimethoxy-5,7-Dihydroxyflavone	Flavonoids	*G. algida*	C_18_H_16_O_7_	Tozhiboev et al. (2011)
58	Isoorientin3′-methyl ether	Flavonoids	*G. veitchiorum*	C_23_H_24_O_11_	Yang et al. (2008)
59	Apigenin	Flavonoids	*G. veitchiorum*	C_15_H_10_O_5_	Yang et al. (2014b)
			*G. algida*		
60	Apigenin-6-C-β-D-glucopyranoside	Flavonoids	*G. veitchiorum*	C_21_H_20_O_10_	Yang et al. (2008), Zou et al. (2010)
61	Apigenin 7-glucoside	Flavonoids	*G. veitchiorum*	C_21_H_20_O_10_	Zong et al. (2015), Li (2017)
			*G. szechenyii*		
62	Isoorientin-4′-diglucoside	Flavonoids	*G. veitchiorum*	C_33_H_40_O_21_	Li et al. (2016b)
63	Isoorientin 4′-O-glucoside	Flavonoids	*G. algida*	C_28_H_32_O_15_	Yang et al. (2013), Li et al. (2016b), Wu et al. (2018)
			*G. veitchiorum*		
			*G. lawrencei*		
64	Isosaponarin	Flavonoids	*G. veitchiorum*	C_27_H_30_O_15_	Li. et al. (2017)
65	Swertisin	Flavonoids	*G. algida*	C_22_H_22_O_11_	Yang et al. (2013)
66	Swertanone	Flavonoids	*G. algida*	C_30_H_48_O	Yang et al. (2013), Li et al. (2016b), Fu et al. (2018)
			*G. veitchiorum*		
			*G. szechenyii*		
67	Quercetagetin-7-O-glucoside	Flavonoids	*G. algida*	C_21_H_20_O_13_	Yang et al. (2014b)
68	Swertiajaponin	Flavonoids	*G. algida*	C_22_H_22_O_12_	Yang et al. (2014a), Fu et al. (2018)
			*G. szechenyii*		
69	Orientin-7-caffeate	Flavonoids	*G. veitchiorum*	C_30_H_26_O_14_	Yang et al. (2014a), Li et al. (2016b), Fu et al. (2018)
			*G. algida*		
			*G. szechenyii*		
70	Mangiferin	Flavonoids	*G. szechenyii*	C_19_H_18_O_11_	Fu et al. (2018)
71	Naringenin	Flavonoids	*G. szechenyii*	C_15_H_12_O_5_	Fu et al. (2018)
72	7-O-feruloylorientin	Flavonoids	*G. veitchiorum*	C_31_H_28_O_14_	Yang et al. (2014a), Li et al. (2016b)
			*G. algida*		
			*G. szechenyii*		
73	Isoscoparin-7-*O*-glucopyranoside	Flavonoids	*G. veitchiorum*	C_28_H_32_O_16_	Li et al. (2016b)
74	Isobellidifolin	Xanthones	*G. algida*	C_14_H_10_O_6_	Tozhiboev et al. (2011)
75	Swerchirin	Xanthones	*G. algida*	C_15_H_12_O_6_	Yang et al. (2013)
76	1,5,8-Trihydroxy-3,4-dimethoxy xanthone	Xanthones	*G. algida*	C_15_H_12_O_7_	Tozhiboev et al. (2011)
77	3-Methoxy-1,5,8-trihydroxy xanthone	Xanthones	*G. algida*	C_14_H_10_O_6_	Tozhiboev et al. (2011)
78	Swertianolin	Xanthones	*G. algida*	C_20_H_20_O_11_	Tozhiboev et al. (2011)
79	3-O-β-D-glucopyranosyl-1-hydroxy-7-methoxy xanthone	Xanthones	*G. algida*	C_20_H_20_O_9_	Bao et al. (2023)
80	3-O-β-D-glucopyranosyl-1,6-dihydroxy xanthone glycoside	Xanthones	*G. algida*	C_19_H_18_O_10_	Tozhiboev et al. (2011)
81	Oleic acid	Organic acids	*G. veitchiorum*	C_18_H_34_O_2_	Huang and Zou (2013), Li (2017)
			*G. szechenyii*		
82	Linoleic acid	Organic acids	*G. algida*	C_18_H_32_O_2_	Li (2013), Yang et al. (2014b)
83	Nonacosylic acid	Organic acids	*G. algida*	C_29_H_58_O_2_	Li (2013)
84	2,3-Dihydroxybenzoic acid ethyl ester	Organic acids	*G. algida*	C_9_H_10_O_4_	Li (2013)
85	Butyl isobutyl phthalate	Organic acids	*G. algida*	C_16_H_22_O_4_	Li (2013)
86	L-Pyroglutamic acid	Organic acids	*G. algida*	C_5_H_7_NO_3_	Bao et al. (2023)
87	Ethyl L-Pyroglutamate	Organic acids	*G. algida*	C_7_H_11_NO_3_	Bao et al. (2023)
88	Daucosterol	Steroids	*G. veitchiorum*	C_35_H_60_O_6_	Zou et al. (2010), Sun (2012), Naonobu. et al. (2015)
			*G. algida*		
			*G. lawrencei*		
89	5,8-dimethoxy-Furan coumarin	Coumarins	*G. algida*	C_13_H_10_O_5_	Han (2012)
90	Acanthoside B	Lignans	*G. veitchiorum*	C_28_H_36_O_13_	(Zong, 2015; Li, 2017)
			*G. szechenyii*		
91	Gentisuric acid	Hydroquinones	*G. szechenyii*	C_9_H_9_NO_5_	Zong (2015)
92	Polygalacerebroside	Amides	*G. veitchiorum*	C_41_H_79_NO_9_	Zou et al. (2010)

### 5.1 Iridoids

Iridoids are primarily distributed among more than 50 plant families and are notably abundant in the Gentianaceae family. Within the genus *Gentiana*, a wealth of iridoids is found, with over 60 species of enol ether terpene glycosides identified to date. Moreover, across various plant species, the tally exceeds 1,000 isolated and identified iridoid compounds. In the realm of Tibetan medicine, the iridoids encompass iridoid glycosides, cleaved iridoid glycosides, strychnine, and several other significant compound groups (Xu et al., 2008). Iridoids play a pivotal role as key active metabolites in gentian plants and serve as characteristic metabolites within Tibetan pharmacopeia. Most of these metabolites are glycosides, with only a few exceptions. Among these, swertiamarin, sweroside, and β-sitosterol enjoy broader distribution in the plant kingdom (Li et al., 2016a; Li et al., 2016c; Olennikov and Chirikova, 2016). Structurally, these compounds exhibit distinctive features, including cyclopentane monoterpene derivative for iridoids. Iridoids glycosides commonly featured C_7_ to C_8_ bond breakage, forming 10 carbon atoms’ compounds. Most of these are in the form of D-glucosides, with C_11_ undergoing oxidation to form carboxylic acids and esters.

Hence, iridoids emerge as the predominant metabolites in Bang Jian, with notable examples including gentiopicroside, sweroside, and swertiamarin. Among these, gentiopicroside assumes a pivotal role and is often utilized as the benchmark metabolite. Consequently, gentiopicroside finds its place in the Sichuan Chinese Materia Medica Standard (2010 edition) and Sichuan Tibetan Materia Medica Standard (2020 edition) as a quality standard for quantifying its content in both blue-flowered and white-flowered gentian species. It is worth noting that szechenyin A, gentizechenlioside A, gentiournosides D, depressine can be regarded as the characteristic metabolites of *G. szechenyii* and *G. stipitata* (Zhong et al., 2014; Ma et al., 2015). Because these characteristic components were isolated and identified only in this species. The next step should be further research on the characteristic metabolites, which can be incorporated into the standard-setting of this variety.

Besides, based on two iridoids metabolites, swertiamarin and gentiopicroside, our group established the first method of HPLC fingerprint analysis between Tibetan medicine Bang Jian, by comparing the fingerprint comparative analysis between different basal sources of Bang Jian (white-flowered gentian, blue-flowered gentian), mixed pseudo-products and traditional Chinese medicine gentian. It showed that the chemical composition of different interspecies differed greatly, which can be used as the basis for their species identification. Among them, the fingerprints of *G. szechenyii* and *G. stipitata* were more similar, with the values of 0.8–0.9, and both of them were less similar to *G. algida* and blue-flowered gentian (Ma, 2014). There are also studies on the quantitative analysis and fingerprinting of loganic acid, swertiamarin and gentiopicroside in the flowers and stems of *G. veitchiorum* and *G. lawrencei*, as well as in the flowers and stems of the same botanical drug, as determined by HPLC. The results showed that the content of gentiopicroside in flowers was higher than that in the whole botanical drug and stems in different species. And the content of loganic acid, and swertiamarin did not have tissue specificity. When evaluating the content of *G. veitchiorum* and *G. lawrencei* with the content of gentiopicroside, the content of different species varied greatly, and the content of the flower part was better than that of the stem and leaf part. The content of the flower parts was better than that of the stem and leaf parts (Ma et al., 2018). This study provides data support for the traditional use of flower parts in Tibetan medicine, provides reference for the standardization of Tibetan medicine and the actual production of Tibetan medicine, and explores the necessity of establishing the standard of ethnomedicine for Daodi botanical drugs.

### 5.2 Flavonoids

Flavonoids have been studied in Chinese medicine and are the main active metabolites in Tibetan medicine. And research has shown that the occurrence of many diseases, such as tumors, cardiovascular and cerebrovascular disease, Alzheimer’s disease, etc., are all associated to varying degrees with damage caused by free radicals (Tozhiboev et al., 2011; Ga et al., 2020). Flavonoids have the ability to scavenge disease-causing free radicals, as well as antibacterial, anti-aging, blood pressure and other health functions, and are a safe, effective, natural oxidant (Tadzhibaev. et al., 1992; Jiao et al., 2012). The basic backbone of flavonoids is C_6_-C_3_-C_6_, and their metabolites can be broadly classified as follows: flavonoids, flavanols, dihydroflavonoids, chalcones, isoflavones, isoflavenes, dihydroisoflavones, etc (Li et al., 2016c). The Tibetan medicine Bang Jian contains a variety of flavonoid metabolites, mainly isoorientin, isoobtusin, sweretin, etc. Most of the flavonoids found in Bang Jian botanical drugs have hydroxyl and phenolic hydroxyl substituents. To date, only seven flavonoids have been isolated from the Bang Jian, and in addition to these compounds, eighteen other flavonoids have been detected or identified by LC-MS and HPLC. Besides, our group has also established the first fingerprint analysis method for the Tibetan medicine Bang Jian based on two flavonoid metabolites, isoorientin and Isovitexin, which can completely differentiate between the *G. szechenyii*, *G. algida* and the other blue-flowered Gentian (Ma L., 2014).

### 5.3 Xanthones

In addition to the flavonoids, a relatively large number of xanthones have been isolated. Xanthones are compounds with an oxygen-containing dibenzo-γ-pyrone heterocyclic scaffold, which are found in the form of mono- or polymethyl ethers and glycosides in this genus (M.M. et al., 2011). The parent nucleus of these compounds is a benzophenanthrone with eight substitutable positions in the parent nucleus. Xanthones exist mainly in the form of O-glycosides and C-glycosides. Xanthones are a class of compounds with a unique structure and are only found in a few families in nature, including Gentianaceae, Guttiferae, Moraceae, Clusiaceae, and Polygalaceae have inhibitory effects on a wide range of bacteria and fungi, and they have diuretic and cardiotonic activities. However, xanthones may not be characteristic metabolites of Bang Jian and the pharmacological activity of such constituents has been less studied.

### 5.4 Triterpenoids

In fact, triterpenoids are not the main active metabolites of Bang Jian, and the content is not high. At present, at least twenty saponin species have been isolated from gentian, and the triterpene saponin elements obtained by isolation are all derivatives of pentacyclic triterpenoids of the 3β-hydroxy oleanane type (Huang and Zou, 2013; Guo et al., 2021). In addition to the distribution of these compounds in the genus Swertia, ursolic acid, carotenosterol, oleanolic acid, and β-sitosterol have also been found in *G. algida* in recent years (Yang et al., 2015). In the 1980s, ursolic acid and oleanolic acid were shown to be effective anti-hepatitis and SGPT-lowering metabolites, and both have certain anti-tumor effects.

### 5.5 Other chemicals

There are other components in Tibetan botanical drug Bang Jian, such as coumarins, alkanols, volatile oils, polysaccharides, and many others (Su, 2004; Zhang, 2009; Xiao et al., 2018). Coniferin was isolated from the roots of Dauri gentian by Wang et al. and 5,8-dihydroxyfuranocoumarin was isolated from white flower gentian (Wang, 2022). Some scholars used gas chromatography-mass spectrometry (GC-MS) and the NIST mass spectrometry library to isolate and identify 83 metabolites from the volatile oil of *G. veitchiorum* flowers, mainly alkanes, with other alcohols, acids, esters, aldehydes, ketones, and phenolic compounds. In addition, it was indicated that Al, Fe, Pb, Cd, Be, Co, Tl, Rb, Sr, Se were the characteristic elements of *G. veitchiorum* (Kang et al., 2022). Moreover, there are extremely significant and significant positive correlations among characteristic elements.

## 6 Pharmacology

As mentioned above, Bang Jian is a traditional Tibetan medicine with a variety of biologically active metabolites and is an ethnic medicine of great research value. Modern research has shown that extracts and isolates from different parts of the Bang Jian are rich in pharmacological activities, including respiratory protection, hepatoprotection, anti-tumor activity, anti-inflammatory activity, antibacterial activity, anti-viral activity, anti-oxidation, etc (Zhang, 2017). The specific pharmacological activities are summarized in Table 4. To date, relevant studies have focused on the anti-inflammatory and respiratory protective effects of *G. veitchiorum* and *G. szechenyii*, for which iridoids and flavonoids are the main active metabolites. Relevant pharmacological studies on other species are less frequent, and most of them are on crude extracts. In addition, research on the individual metabolites, including the identification of compounds, mechanisms of action, and molecular signaling pathways, have been lacking in the literature in recent years. Therefore, there is still a need for more in-depth and comprehensive studies on the material basis and mechanism of action of the pharmacological effects.

**TABLE 4:** Summary of the pharmacological activities of Bang Jian.

Pharmacological activity	Test substance/part	Test system	Tested method	Positive drug	Results	References
Respiratory system protection	*G. veitchiorum* was extracted by ethanol maceration, and the extract was concentrated and extracted by petroleum ether, chloroform, ethyl acetate, and n-butanol to obtain five products with different polarities	Chronic bronchitis model in mice due to ammonia stimulation	i.g 4 g/kg	GuiLongKeChuanNing Capsule	1) Compared with the model group, T-AOC activity in lung tissue of the n-butanol and aqueous layer groups was significantly higher (*p* < 0.01); SOD activity in lung tissue of the ethyl acetate and n-butanol groups was significantly higher (*p* < 0.05 or *p* < 0.01), and the serum levels of TNF-*α*and IL-10 of the ethyl acetate and n-butanol groups were significantly reduced (*p* < 0.05 or *p* < 0.01)	Geng et al. (2010)
					2) HE staining Pathological sections showed that the airway damage of chronic bronchitis in mice in the ethyl acetate and n-butanol extract treatment group was significantly improved after treatment with each polar metabolite of *G. veitchiorum* Hemsl	
	*G. veitchiorum* particles	mice inhaled NH_3_ with YLS-8A equipment to induce the chronic bronchitis	i.g 4, 2, 1 g/kg	FuFangQiGuanYan Tablets	1) Compared with the model group, the contents of MDA in lung homogenate were decreased significantly and the contents of SOD were increased significantly in *G. veitchiorum* treatment groups	Hou et al. (2008), Hou (2011)
					2) HE staining showed the alleviation of injury from chronic bronchitis treated with *G. veitchiorum* particles	
	*G. veitchiorum* particles	the chronic asthmatic model was established with the ovalbumin sensitization and repeated inhalation of ovalbumin aerosols	i.g 0.33 g/kg	Dexamethasone	1) The difference of the dimensions, such as the positive staining of AB-PAS/Pbm, the positive staining of Bcl-2/Pbm between group B and group C were significant (*p* < 0.01), Aep/Pbm in B group was significantly higher than that in group C (*p* < 0.01), but no difference with group D	Zhang et al. (2009a)
					2) A close correlation between the positive staining of AB-PAS and the Bcl-2 content (r = 0.671, *p* < 0.001), and the r = 0.784 between the Aep/Pbm and the positive staining of Bcl-2 was significant, too	
	*G. veitchiorum* granules	30 male SD rats normal control group, 30 Gy irradiation group and Lanyuzan granules group	CT imagines and histopathological changes	—	1) The rats’ lung CT images changed at different time periods and especially after 30 damaging showed fibrosis changes significantly. There was significant difference between Lanyuzan granules group and the 30 Gy irradiation group in pulmonary fibrosis imaging changes	Shi et al. (2016)
					2) Histopathology examinations showed rat lung tissue in Lanyuzan granules group were significantly alleviate than the 30 Gy irradiation group. Statistics results shows that there were significant different in hydroxyproline content between 30 Gy irradiation group and 30 Gy irradiation treatment group which accepted irradiation treatment with Lanyuzan granules (8 and 10 days b Post irradiation *p* < 0.01)	
Hepatoprotective effect	*G. veitchiorum*	Hepatic fibrosis induced by DMN	i.g, 2 g/kg, 1/d, 60 days	Predniso-ne Acetate Tablets	Regulation of lipid peroxidation *in vivo* and inhibition of hepatic stellate cell (HSC) activation	Li et al. (2008b)
	*G. veitchiorum* granule	Hepatic fibrosis induced by DMN	i.g, 2 g/kg, 1/d, 60 d	IFN-γ	1) In liver fibrosis model group, serum concentrations of ALT and AST were significantly higher, and concentration of ALB was significantly lower (*p* < 0.01), while significant increase in *α*-SMA levels and decrease in MMP-2 activity were observed compared with the control group (*p* < 0.01)	Fang (2009)
					2) Decrease ALT and AST serum concentrations, significantly increased ALB concentration, lowered *α*-SMA expression and elevated MMP-2 activity in liver tissue, were observed in Lanyuzan granule treatment group compared with model group (*p* < 0.01)	
					3) No notable difference was observed between low dosage group and model control group in MMP-2 activity. The pathological sections showed hepatic fibrosis was alleviated markedly	
	*G. veitchiorum* Methanol Extracts	CCl_4_-induced acute liver injury	100, 200, 400 mg/kg	Silymarin	1) The levels of MAO and MDA were reduced and the levels of SOD and GSH were increased in the liver tissues of rats in the *G. veitchiorum* group	Zhang et al. (2014)
					2) Improvement in hepatic fibrosis was observed by HE and Masson staining	
Anti-inflammatory activity	*G. szechenyii* aqueous and ethanolic extracts	lene-induced acute ear swelling in mice and capillary permeability in the mouse abdomen	i.g 3.0, 1.5,0.8 g/kg, 14 days	—	1) The flowers of Gentiana alba also have good anti-inflammatory properties	Yang et al. (2010)
					2) Alcoholic extracts are significantly more effective than aqueous extracts	
	n-butanol, ethyl acetate, petroleum ether exacts of *G. szechenyii*	25% ammonia chemical stimulation to the rat acute pharyngitis model	i.g 0.5 g/kg, once/d, 7 days	QingHouLiYan Particle	The rats throat mucosa of model control group appeared obvious pathological changes, compared with model control group, *G. szechenyii* different polar parts group of rats with acute pharyngitis had different degrees of improvement, in which the pharyngeal lesions were repaired obviously, which could reduce the levels of IL-10, TNF-*α* in rat serum (*p* < 0.01)	Zhang et al. (2018)
	SanWeiLongDanHua Tablet (*G. szechenyii*)	Establishment of an allergic asthma model by intraperitoneal injection of ovalbumin sensitisation and nebulised inhalation provocation	High dose: 3.43 g/kg, medium dose 1.71 g/kg, low dose 0.86 g/kg groups	Dexamethasone	1) The latency of asthma in groups of the high, middle and low doses of Sanwei Longdanhua tablets were significantly longer than that of model group (*p* < 0.01)	Zhao et al. (2018)
					2) Compared with the model group, the levels of IL-4 and IFN-γ were significantly increased in dexamethasone group and Sanwei Longdanhua tablet high, middle, and low doses groups (*p* < 0.05)	
					3) The bronchial epithelial cells proliferated in model group with more mucus secretions in bronchial lumen, pulmonary interstitial edema, and increased cellular metabolites. A small amount of inflammatory exudate was found in the bronchial lumen of dexamethasone group and Sanwei Longdanhua tablet group	
Antibacterial activity	Ethanol, chloroform, n-butanol, distilled water extracts of *G. veitchiorum*	Methicillin-resistant *Staphylococcus aureus* (MRSA), methicillin-sensitive *Staphylococcus aureus* (MSSA) strains	Vitro: bouillon dilution method	YinHuangCapsule	The n-butanol fraction showed the highest inhibitory effect, followed by ethanol, aqueous layer fraction	Liu et al. (2011)
			Vivo:Establishment of a mouse infection model using intraperitoneal MRSA injection method			
	Extracts of *G. veitchiorum*	*Staphylococcus aureus* and *Escherichia coli*	Bacterial ring method	—	*Staphylococcus aureus* had the most pronounced inhibitory effect with a MIC value of 99.3 mg/L, followed by *Escherichia coli* (MIC value 225.5 mg/L) and *Candida albicans* (MIC value 126.8 mg/L), and *Salmonella* and *Shigella* dysenteriae	Geng et al. (2019)
	Petroleum ether, ethyl acetate, n-butanol extracts of *G. algida*	*Escherichia coli*, *Staphylococcus aureus*, *Streptococcus pneumoniae*, *Pseudomonas aeruginosa*, *Bacillus licheniformis*	Oxfordshire cup method	Penicillin/Streptomycin	1) The ethyl acetate and n-butanol extracts of *G. algida* were found to be bacteriostatic against five species of bacteria, and their bacteriostatic effect increased with the increase in the concentration of the extracts	Han (2012)
					2) Whereas the petroleum ether extract of *G. algida* showed antibacterial effect only against *Staphylococcus aureus*, *Pseudomonas aeruginosa*, and *Bacillus licheniformis*	
Antitumor activity	Oleanolic acid and ursolic acid of *G. algida*	Human leukaemia cells	—	—	Oleanolic acid and ursolic acid may also enhance immunity to splenoma cells by promoting the body’s immune organs	Huang and Zou (2013)
	3-Filicene, arborinone, boehmerol, carotenoside, 3β-acetoxy-28-hydroxy-12-en-ursane and swertisin from *G. algida*	Human cervical cancer Hela cells	MTT	—	The activity of isoscoparin was the strongest, with an IC_50_ value of 45.75 μg/mL. Boehmerol was the next most active, with an IC_50_ value of 51.15 μg/mL. Ursolic acid, 3*β*-acetoxy-28-hydroxy-12-en-ursane, arborinone with an IC_50_ value of 115.52, 117.01, 235.25 μg/mL, respectively. 3-Filicene was the worst with an IC_50_ value of 280.07 μg/mL	Han (2012)
	Patulitrin, oscoparin, swertisin, Swertiajaponin of G. algida	Hepatocellular carcinoma (HepG2) cells	MTT	—	Oscoparin has high cellular activity with an IC_50_ of 47.25 g/mL	Khobrakova et al. (2017)
Antiviral activity	Aqueous decoction and n-butanol, ethyl acetate, chloroform extracts of *G. veitchiorum*	Vitro: Hep-2 cells CPE and MTT	i.g	Penicillin/Streptomycin	Vitro: Compared with the virus control group, the viability of RSV-infected cells increased to 77.3% ± 7.1%, 8.4% ± 6.5%, 50.4% ± 3.8% and 47.5% ± 5.2% with the decoction of *G. veitchiorum* and the extracts of n-butanol, ethyl acetate and chloroform, respectively; the inflammatory reaction was significantly milder in the treatment group than in the virus control group	Wei (2011), Wei et al. (2011)
		Vivo: Respiratory syncytial virus (RSV) infected	0.5 mL, 7 days		Vivo: Compared with the virus control group, the inflammatory reaction of the *G. veitchiorum* decoction treatment group was significantly milder than that of the virus control group	
	Chinese gentian	The anti-RSV effect of Chinese Gentian in HeLa cell culture was observed by means of the cytopathic effect inhibition assay	—	Bingduzuo Injection	In HeLa cell culture, Chinese Gentian was found to be an inhibitor of RSV in a concentration-dependent manner, and the TC_50_ was 15.25 mg/mL, and the EC_50_ was 1.07 mg/mL, and the TI was 14.25	Li and Li (2006)
Antioxidant	Isoorientin, isoscoparin, gentiournosides E, szechenyin B and gentiournosides A isolated from *G. szechenyii*	HPLC-DPPH-MS/MS	—	L-Ascorbic acid	The strongest antioxidant activity was found in gentiournosides E, and the weakest was found in szechenyin B	Zhang et al. (2022)
	Apigenin isolated from *G. veitchiorum*	DPPH	—	—	Not only does it reduce the production of reactive oxygen species, but it also regulates cholesterol metabolism	Dou et al. (2020)
	*G. veitchiorum* particles	DPPH	—	—	Enhance the vitality of antioxidant enzymes in the body, regulate oxidation and antioxidant balance	Hou (2011)

### 6.1 Protection of the respiratory system

Bang Jian has a long history of medicinal use, with the effects of detoxification and the treatment of various kinds of fever, laryngitis, and heat closure. As a commonly used in Tibetan medicine, *G. szechenyii* is used as the main raw material in many compound preparations of Tibetan medicines under the State Pharmaceutical Approval Number (Table 5), which is used for relieving coughs and asthma, and treating respiratory system diseases such as acute and chronic bronchitis clinically. In addition, in the Tibetan areas of Qinghai province and Tibet of China, *G. veitchiorum*, *G. szechenyii*, and *G. algida* are often administered separately to treat respiratory diseases such as bronchitis (Yang, 1991). At present, research on the respiratory system mainly exists in two aspects: chronic bronchitis and pulmonary fibrosis.

**TABLE 5:** Clinical application of preparations of Bang Jian.

No.	Name	Main compositions	Clinical application	Standards	References
1	SanWeiLongDanHuaPian	*G. szechenyii,* Licorice, honey balm	Clears heat and moistens the lungs. For heat and asthma of the lungs and pharyngitis	Ministry of Health Drug Standard Tibetan Medicine Book I	Chinese Pharmacopoeia Commission (1995)
2	ShiWeiLongDanHua Keli	*G. szechenyi, Rhododendron anthopogonoides*, Licorice, *Corydalis hendersonii*, *Fritillaria cirrhosa*, Xiaobopi, Jidanshen, *Phlomoides younghushandii*, *Inula racemosa, Przewalskia tangutica*	Relieves cough and asthma, clears heat, and dispels phlegm	Ministry of Health Drug Standard Tibetan Medicine Book I	Chinese Pharmacopoeia Commission (1995)
3	ShiWeiLongDanHua Jiaonang	*G. szechenyii, Rhododendron anthopogonoides*, Licorice, *Corydalis hendersonii*, *Fritillaria cirrhosa*, Xiaobopi, Jidanshen, *Phlomoides younghushandii*, *Inula racemosa, Przewalskia tangutica*	Relieves cough and asthma, clears heat, and dispels phlegm	Ministry of Health Drug Standard Tibetan Medicine Book I	Chinese Pharmacopoeia Commission (1995)
4	ShiwuWeiLongDanHua Jiaonang	*G. szechenyii*, *Santalum album*, *Terminalia chebula*, *Terminalia billerica*, *Phyllanthus emblica*, Shihuihua, Muxiang, guangzao, *Eugenia caryophyllata*, *Myristica fragrans*, *Tinospora sinensis*, *Aquilaria sinensis*, Baxiaga, Wujingji, Licorice	Cleanses heat and regulates the lungs, relieves cough, and dissolves phlegm. For bronchitis and emphysema, cough and asthma, hoarseness, and huskiness	Ministry of Health Drug Standard Tibetan Medicine Book I	Chinese Pharmacopoeia Commission (1995)
5	ShiwuWeiLongDanHua Wan	*G. szechenyii*, *Santalum album*, *Terminalia chebula*, *Terminalia billerica*, *Phyllanthus emblica*, Shihuihua, Muxiang, guangzao, *Eugenia caryophyllata*, *Myristica fragrans*, *Tinospora sinensis*, *Aquilaria sinensis*, Baxiaga, Wujingji, Licorice	Cleanses heat and regulates the lungs, relieves cough, and dissolves phlegm. For bronchitis and emphysema, cough and asthma, hoarseness, and huskiness	Ministry of Health Drug Standard Tibetan Medicine Book I	Chinese Pharmacopoeia Commission (1995)

Firstly, chronic bronchitis is one of the most common respiratory diseases, which mainly refers to the chronic non-specific inflammation of the respiratory system trachea, tracheal mucosa, and surrounding tissues (Lahousse et al., 2017). It is clinically characterized by cough, sputum, or wheezing, and can be complicated by emphysema, pulmonary hypertension, and pulmonary heart disease (Raju et al., 2016). At present, antibacterial drugs are mainly used in clinical treatment. However, the toxic side effects are serious, and it is easy to produce drug resistance. Long-term use of antibacterial drugs can also cause a bacterial imbalance, increasing the possibility of developing other diseases.

The second is pulmonary fibrosis, which is also a common chronic, interstitial lung disease in respiratory diseases. It is characterized by progressive dyspnea, which may further develop respiratory failure and lead to death (Mathai and Schwartz, 2019; Liu et al., 2022). Currently, the treatment options for pulmonary fibrosis are also very limited, and the main clinical tools are anti-pulmonary fibrosis drugs and lung transplantation. However, the clinical prognosis is poor, the incidence rate is increasing year by year, which seriously threatens human health and life, and the clinical lack of effective treatment methods.

Due to the advantages of multi-targets, multiple efficacy and low side effects, the research of traditional Chinese medicine in treating pulmonary fibrosis has attracted wide attention. Currently, according to the results of existing studies, the ethyl acetate site and the n-butanol site are the effective sites for the treatment of lung diseases by the *G. veitchiorum*. Some studies have shown that *G. veitchiorum* extract has a certain effect on the treatment of chronic bronchitis mice. The results showed an increase in T-AOC and SOD activity and a decrease in serum TNF-α and IL-10 levels in the lung tissue of mice in the ethyl acetate and n-butanol groups. Pathological sections showed significant improvement in the symptoms of chronic bronchitis airway damage in mice. Similarly, several studies have investigated the therapeutic effect of nebulised inhalation of *G. veitchiorum* in a model of TGF-β1-induced pulmonary fibrosis. It can be considered to have an antagonistic effect on the development of pulmonary fibrosis induced by TGF-β1. From the HPLC of the alcoholic extracts of *G. veitchiorum*, we could find that the substances in the ethyl acetate and n-butanol fractions have a higher likelihood of containing glycosides, and one of the substances, identified by HPLC comparison, is gentiopicroside. Gentiopicroside has been proven to have anti-inflammatory activity in previous studies, so it may also be one of the active metabolites in the treatment of respiratory diseases by *G. veitchiorum*.

In addition to the examination of the activity of *G. veitchiorum*, there is also the study of *G. veitchiorum* granules. And *G. veitchiorum* granules have better efficacy in chronic bronchitis. After the administration of *G. veitchiorum* granules, both SOD and MDA were restored to normal levels in mice suffering from chronic bronchitis. It may be possible to treat chronic bronchitis through antioxidant damage, enhancement of the organism’s antioxidant enzyme activity, and regulation of the oxidative and antioxidant balance (Hou, 2011). In addition, in mice with pulmonary fibrosis, the levels of SOD and HP in lung tissue were measured and showed good control and relief. The reason may be the inhibition of airway inflammation and airway reconstruction in chronic asthma mice, and the mechanism may be related to the inhibition of TGF-*β* expression (Zhang, 2009). It forms a certain foundation for future in-depth research on the active metabolites of *G. veitchiorum* in the treatment of lung diseases.

### 6.2 Hepatoprotective effect

There are many causes of liver system disorders, among which liver fibrosis is a common liver disease. Liver fibrosis is a process of excessive repair after liver parenchymal injury in chronic liver disease, which is mainly characterized by excessive deposition of extracellular matrix, and further develops into cirrhosis or even liver cancer, which is an important public health problem threatening human health. At present, there is no effective medicine for liver fibrosis in Western medicine, and in recent years, studies have found that traditional Chinese medicine can control or even reverse liver fibrosis, which is worthy of in-depth exploration. It has been found that *G. veitchiorum* can prevent DMN-induced hepatic fibrosis by regulating lipid peroxidation and inhibiting the activation of hepatic stellate cells (Li et al., 2008a; Li, 2008). Through *in vivo* animal experiments, it was found that the methanol extract of *G. veitchiorum* had a protective effect against carbon tetrachloride-induced liver injury in mice, which was partly attributed to its ability to scavenge carbon tetrachloride-associated free radicals via scavenging (Li et al., 2008b; Zhang et al., 2014). Besides, except the normal group, all the rats were injected with dimethyl nitrosamine to establish the fibrosis model. And the results showed that compared with the model group, the level of MAO and MDA decreased in the liver tissues of rats in the G. veitchiorum group. It was found that MAO and MDA levels were reduced and SOD and GSH levels were increased in the liver tissues of rats in the G. veitchiorum group compared with the model group, and the degree of hepatic fibrosis was improved as observed by HE and Masson staining (Li et al., 2008a).

### 6.3 Antitumor activity

Malignant tumors are major diseases that pose a serious threat to human health. Studies have reported that as of 2020, malignant neoplasms are responsible for more than 10 million deaths, accounting for one-sixth of global deaths (Sung et al., 2021). Among them, breast cancer, lung cancer, liver cancer, colon cancer, and cervical cancer have higher incidence rates (Arranja et al., 2017). At present, traditional Chinese medicine and ethnomedicine have special advantages and remarkable efficacy in the treatment of tumors, and their main anti-tumor pathways include inhibition of tumor cell proliferation, differentiation, and induction of apoptosis of hepatoma cells (Cheng et al., 2020; Xu et al., 2020).

The current research on the antitumor activity of Bang Jian is mainly focused on the study of flavonoids, iridoids, and triterpenoids, which are the monomer metabolites in *G. algida*. It mainly targets leukaemia and human cervical cancer cells and hepatocellular carcinoma cells. Specifically, inhibition of tumor cell proliferation and unlimited proliferation of malignant tumor cells are closely related to the activation of telomerase, which is a ribonucleoprotein complex that can sustain the proliferation of malignant cells, and there is no activated telomerase in normal tissues. It was found that oleanolic acid and ursolic acid in *G. algida* can be used in human leukemia cells for a period of time, the activity of telomerase within the malignant cells is reduced, thus contributing to the decline in cell growth rate apoptosis rate increases. Oleanolic acid and ursolic acid can significantly inhibit the growth of human cervical cancer cells, but also through the promotion of the body’s immune organs such as the growth of the spleen and lymphocyte proliferation to improve the immunity to splenic tumor cells and enhance the body’s anti-splenic tumor immune response to achieve the effect of anti-tumor. The compounds 3-Filicene, arborinone, boehmerol, carotenoside, 3β-acetoxy-28-hydroxy-12-en-ursane and swertisin from *G. algida* (Han, 2012). And these have different degrees of anti-tumor activity against human cervical cancer Hela cells. Similarly, some scholars found that four flavonoids, including patulitrin, oscoparin, swertisin, and swertiajaponin, were isolated from *G. algida*. The cytotoxicity of four flavonoids on the growth of HepG2 cells was investigated, and the results showed that oscoparin possessed high cellular activity with an IC_50_ of 47.25 g/mL (Yang et al., 2014a).

### 6.4 Anti-inflammatory activity

Inflammation is involved in many complex diseases and organismal disorders, including autoimmune diseases, metabolic syndrome, neurodegenerative diseases, cardiovascular diseases, and cancer (Felger and Treadway, 2017). Studies on the anti-inflammatory effects of Bang Jian mainly include the pharmacological studies of different solvent extracts of *G. algida*, *G. szechenyii*, and *G. veitchiorum* on model animals and the prediction of their mechanisms of action (Li et al., 2001; Chen et al., 2008; Yang et al., 2010). It was found that the aqueous and ethanol extracts of *G. algida* had inhibitory effects on xylene-induced acute ear swelling and abdominal capillary permeability in mice in both the medium and high dose groups, indicating that *G. algida* has anti-inflammatory activity (Yang et al., 2010). It was also found that the alcoholic extract of *G. purdomii* had anti-inflammatory effects at a certain concentration through mouse ear swelling experiments and granuloma experiments (Ga et al., 2020). It was experimentally confirmed that *G. szechenyii* extracts had ameliorative effects on ammonia-induced acute pharyngitis model in rats, and its mechanism of action may be related to the reduction of TNF-α, IL-1β, and IL-6 levels (Liu et al., 2018). Our group also confirmed the anti-inflammatory effects of *G. szechenyii* and *G. veitchiorum* through the experiments of ear swelling and foot swelling in mice in the previous period. And it was concluded that the combined anti-inflammatory and antibacterial efficacy of the flavonoid metabolites of *G. szechenyii* was stronger than that of the iridoids parts. The combined efficacy of iridoids such as loganic acid, gentiopicroside and sweroside in *G. veitchiorum* was found to be stronger than that of the flavonoids such as isoorientin, isoscoparin-2″-β-D-glucopyranoside and isoscoparin.

### 6.5 Antibacterial activity

Antibiotic resistance is a growing medical problem. The Centers for Disease Control and Prevention estimates that drug-resistant bacteria cause 23,000 deaths and 2 million illnesses in the United States each year (Khobrakova et al., 2017). Therefore, it is urgent to find new antibacterial drugs that are effective against drug-resistant strains of bacteria. As a natural active product, Bang Jian has antibacterial activity with iridoids, flavonoids, triterpenoids, and xanthones, etc. Among them, the strongest antibacterial activity is xanthones and iridoids, such as demethylbellidifolin, mangiferin, swertiamarin and gentiopicrin (Lu et al., 2018). The research about the Tibetan medicine Bang Jian found that the extracts (ethanol, chloroform, n-butanol, distilled water) of different polarities of *G. veitchiorum* had different degrees of bacteriostatic effects on methicillin-resistant *Staphylococcus aureus* (MRSA) and methicillin-sensitive *S. aureus* (MSSA) strains, among which the n-butanol fraction had the strongest bacteriostatic effect, followed by ethanol and aqueous-layer fractions (Liu et al., 2011). In addition, it was found that the extract of *G. veitchiorum* had inhibitory effects on both *S. aureus* and *Escherichia coli* (Geng et al., 2019). Moreover, the solid-liquid extraction method was used to extract the active metabolites from the *G. veitchiorum* flowers, and the results of *in vitro* bacteriostatic experiments showed that the extract of *G. veitchiorum* flowers had the most obvious inhibitory effect on *S. aureus*, with a MIC value of 99.3 mg/L, and the second most obvious inhibitory effect on *E. coli* (MIC value 225.5 mg/L) and *Candida albicans* (MIC value 126.8 mg/L), and the inhibitory effect on *Salmonella* and *Shigella dysenteriae* was insignificant (MIC value 126.8 mg/L). The inhibitory effects on *Salmonella* and *S. dysenteriae* were not significant (Geng et al., 2020). And Han et al. found that the ethyl acetate isolates of *G. algida* had good inhibitory activity against five curative bacteria including *E. coli*, *S. aureus*, *Streptococcus pneumoniae*, *Pseudomonas aeruginosa* and *Bacillus licheniformis* (Han, 2012).

### 6.6 Antiviral activity

*G. veitchiorum* decoction and n-butanol, ethyl acetate, and chloroform extracts inhibited respiratory syncytial virus (RSV) *in vitro* and *in vivo*. It was found that the decoction of *G. veitchiorum* was more effective than n-butanol, chloroform, and ethyl acetate extracts in inhibiting the entry of the virus into the cells. Meanwhile, pathological sections of mice infected with RSV treated by gavage with the decoction of *G. veitchiorum* showed a significant reduction of inflammatory exudation in the group of mice compared with that in the control group. Immunofluorescence experiments showed that the fluorescence brightness and the number of particles were reduced in the drug-treated sections, indicating that *G. veitchiorum* showed certain effects in both anti-RSV value-added and anti-RSV-induced inflammation (Wei et al., 2011). Moreover, the aqueous extract of Gentian, RG2-1 and RG3-1, the effective antiviral parts of Gentian, inhibited respiratory syncytial virus *in vitro*, and the quantitative effect relationship was obvious.

### 6.7 Antioxidant

Oxidative stress predisposes to lipid membrane peroxidation, which damages membrane integrity and leads to cell death, causing major diseases such as atherosclerosis, diabetes, cancer, and respiratory diseases (Hayes et al., 2020; Griendling et al., 2021). Studies have shown that flavonoids and iridoids are the main antioxidant active metabolites of Bang Jian species. The main studies have focused on the evaluation of antioxidant properties *in vitro*. Bang Jian botanical drugs are mainly used in the treatment of the respiratory system.

Since the abnormal activation of macrophages in upper respiratory tract infections is closely related to reactive oxygen species (ROS) signaling in cells, and the mechanism of cellular damage caused by viral infection and hypoxia is also related to oxidative damage caused by the accumulation of reactive oxygen species, treatment is often supplemented with antioxidants. It has been found that antioxidants may prevent hypoxic tissue damage and reduce viral cellular damage by inhibiting ROS signaling-dependent macrophage overactivation. Therefore, HPLC-DPPH-MS/MS was used for rapid screening and identification of antioxidant active metabolites in *G. szechenyii*. The HP online antioxidant screening system was used to carry out the screening of active compounds, the active products were separated and structurally identified using preparative chromatography. Besides, the antioxidant capacity of the five main active metabolites was determined using DPPH. The results showed that five antioxidant active compounds were screened from *G. szechenyii*, namely, isoorientin, isoscoparin, gentiournosides E, szechenyin B and gentiournosides A. In addition, gentiournosides E had the strongest antioxidant activity and szechenyin B was the weakest of the five compounds (Zhang et al., 2022). Similarly, it was found that apigenin, extracted and isolated from *G. veitchiorum*, is a flavonoid compound with antioxidant activity that not only reduces the production of reactive oxygen species but also regulates cholesterol metabolism (Dou et al., 2020). Therefore, tapping the natural antioxidant active metabolites in clinically applied Chinese and Tibetan botanical drugs for the treatment of upper respiratory tract infections is expected to produce desirable adjuvant therapeutic effects on respiratory system diseases and provide new ideas for clinical treatment.

## 7 Discussion

*Gentiana i*s an annual or perennial botanical drug. In Gentianaceae, the genus has the largest number of plant species, *Gentiana* plants can be used for ornamental, medicinal and other purposes, and most of them are traditional Tibetan medicine. Tibetan medicine Bang Jian is the generic name for a variety of medicinal plants in the genus *Gentiana*, which is representative of one of the commonly used medicines in Tibetan medicine. It is one of the commonly used medicines in Tibetan medicine. It has the efficacy of treating poisonous diseases, various kinds of fever, laryngitis, heat paralysis and so on. The efficacy is exact and widely used.

This review provides an overview of the botanical characteristics, traditional ethnomedicinal applications, chemical composition, and pharmacological properties of Bang Jian. Tibetan medicine boasts a rich historical lineage, utilizing various parts of botanical drugs, each yielding distinct therapeutic benefits. Among the frequently employed the whole grass in Tibetan medicine, Bang Jian is predominantly administered in the form of dried flowers. These flowers are readily available and renowned for their remarkable therapeutic efficacy. Multiple studies have corroborated the global utilization of various metabolites of Tibetan medicine Bang Jian in ethnobotanical practices. Pharmacological research has shed light on the biological activities of Tibetan medicine Bang Jian, enriching our understanding of its medicinal properties. Despite significant advancements in Bang Jian research, there are still some gaps in our knowledge, particularly in uncovering novel discoveries in this field.

Currently, the complexity of the origins of the Tibetan medicine Bang Jian, according to botanical drug records are used to classify type, according to the flower color is divided into white, black, blue, miscellaneous. The classification is confusing, and the varieties are named differently, which is not conducive to the standardization of the varieties and the accuracy of the medication. It is necessary to classify the varieties through resource science, molecular biology and other methods, and molecular biology is an effective means of identifying species that are easily confused with their relatives, and genomics, transcriptomics and gene barcode identification can be considered, which makes it possible to provide more reasonable and standardized guidance for clinical use of medicines.

Besides, Bang Jian botanical drugs are primarily utilized within ethnic medicine, with a majority of studies conducted domestically and limited involvement from foreign scholars. Through the summary of the chemical composition of Bang Jian, it was found that there were some differences in the chemical composition between the white flower Gentian and the blue flower Gentian. The iridoids of gentiopicroside and swertiamarin are the main characteristic metabolites of blue flowers. However, szechenyin A, gentizechenlioside A, depressine, and gentiournoside D were the main iridoid metabolites identified in white flowers, while these metabolites are not identified in blue-flowered gentian. It is well known that differences in ingredients can lead to differences in efficacy. Therefore, it is necessary to combine the characteristic metabolites with pharmacodynamics research in the next step to find pharmacodynamic markers. It further provides guidance for clinical medication.

Furthermore, current pharmacological research into the effectiveness of Tibetan botanical drugs is predominantly focused on just two or three species, neglecting other valuable species. And the research on the pharmacological activity of mainstream species is still in the primary stage. The main research is in some simple pharmacodynamic experiments. Expanding the scope of research on Bang Jian represents a logical next step. It is crucial to highlight that research into the metabolites and mechanisms of action of Tibetan medicine Bang Jian is somewhat superficial. Pharmacological investigations often center on crude extracts, with limited exploration of the effects of individual metabolites. Therefore, it is necessary to carry out a comprehensive study on the chemical composition of Bang Jian and the differences between different medicinal parts. The pharmacological effect studies of single compound and clinical efficacy studies should be strengthened for a more rational utilisation of Bang Jian.

Tibetan medicine Bang Jian is not only a medicinal plant, but also a very ornamental flowering plant, which occupies an important position in the vegetation of the plateau and in the system of Tibetan medicine. However, due to the wide application of Tibetan medicine and its unique therapeutic efficacy in recent years, the number of wild resources has been declining year by year, and the regeneration capacity of the species involved has been decreasing year by year in the harsh growing environment of the plateau. In order to effectively solve the problem of the resource quantity of Bang Jian, in addition to the research on the origin, pharmacological activity and chemical composition of Bang Jian species, people should be called upon to protect its wild resources and carry out the related artificial nursery technology. While expanding the resources of Bang Jian, it can also provide an effective reference for the development of the local tourism industry, so as to improve the economic and ecological benefits. Also, to preserve these invaluable resources and fully harness their potential, there is an urgent need to bolster research efforts into the pharmacological foundation and clinical applications of Bang Jian.

## 8 Conclusion and perspectives

This review provides a comprehensive account of the traditional uses, botanical characteristics, phytochemistry, and pharmacological activities of the Bang Jian group of botanical drugs in Tibetan medicine. The Tibetan medicine Bang Jian botanical drug have a long recorded history and are receiving increasing attention as a commonly used botanical species in traditional Tibetan medicine. In summary, in terms of traditional medicinal use, it is interesting to note that unlike the traditional Chinese medicine of gentian, which is administered from the root, traditional Tibetan medicine usually uses the dried flowers of Bang Jian in medicine. It is mainly used to treat fever, laryngitis, and pyrexia. The main active metabolites of Bang Jian are iridoids, flavonoids, triterpenoids, and xanthones. A total of 92 compounds have been isolated and identified, including 18 iridoids, 26 flavonoids, 29 triterpenoids, 7 xanthones, and 12 other compounds. According to modern pharmacological studies, Bang Jian has a wide range of pharmacological activities. These include respiratory protection, hepatoprotection, antitumor, anti-inflammatory, antibacterial, antiviral, and antioxidant activities. It provides a reference for the further development and utilization of Tibetan medicine Bang Jian.

Despite the extensive medicinal value of Bang Jian as a traditional Tibetan medicine. However, there are some limitations as well. Firstly, at present, Bang Jian botanical drugs contain a rich variety of metabolites, such as iridoids, flavonoids, triterpenoids and so on, which are found in the flowers and above-ground parts of the Bang Jian group of medicinal plants. However, the current quality standard of Bang Jian only uses gentiopicroside and isoorientin as indicator metabolites. More importantly, it is incomplete to measure the quality of botanical drugs only by a single or two indicator metabolites. Secondly, there is a wide range of pharmacological studies, including anti-inflammatory, antitumor, antiviral and antioxidant aspects, but most of them are focused on three species, *G. veitchiorum*, *G. algida* and *G. szechenyii*, with fewer studies on the pharmacological activities of other species. In addition, research on the pharmacological activity of Bang Jian botanical drug is still at a preliminary stage. The pharmacological substance basis of the Bang Jian botanical drug is not clear enough. Most of the studies were on crude extracts, and there were fewer studies on single pharmacodynamic components, as well as studies on pharmacodynamic mechanisms still need to be further explored and expanded.

Therefore, in order to further protect the resources and develop their medicinal value, it is necessary to strengthen the research on the pharmacological material basis and clinical application of Bang Jian botanical drugs. Specifically, it is necessary to carry out research on the phytochemistry of the various parts of Bang Jian botanical drugs of Tibetan medicine and to strengthen the research on the pharmacological effects of monomer phytochemistry, pharmacodynamic mechanism and clinical efficacy. In order to lay a certain foundation for the more reasonable and effective use of the plant resources of Tibetan medicinal botanical drugs, it is necessary to carry out research on the chemical composition of each part of Tibetan medicinal drugs. Then, in order to ensure the safety and efficacy of clinical use of drugs. It is necessary to establish a more perfect theoretical system of quality standards and control of traditional Chinese medicine and Tibetan medicine through multi-indicator quantitative composition analysis combined with pharmacodynamic experiments. In addition, further development of applications other than medicinal plants of Bang Jian can also be carried out. It is known through pharmacological studies that Bang Jian botanical drugs also have anti-inflammatory and antioxidant effects, which can be considered to be developed into healthcare products such as tea. In addition, with its bright color and graceful appearance, it has high ornamental value. It can be domesticated and cultivated as an ornamental plant. In summary, we can make fuller and more comprehensive use of Bang Jian and realize its great potential.
