# Supplementary figures and images for: Tibetan medicine Bang Jian: a comprehensive review on botanical characterization, traditional use, phytochemistry, and pharmacology
Source: Front Pharmacol. 2023 Dec 14;14:1295789. doi: 10.3389/fphar.2023.1295789 (PMC10757618; doi:10.3389/fphar.2023.1295789)

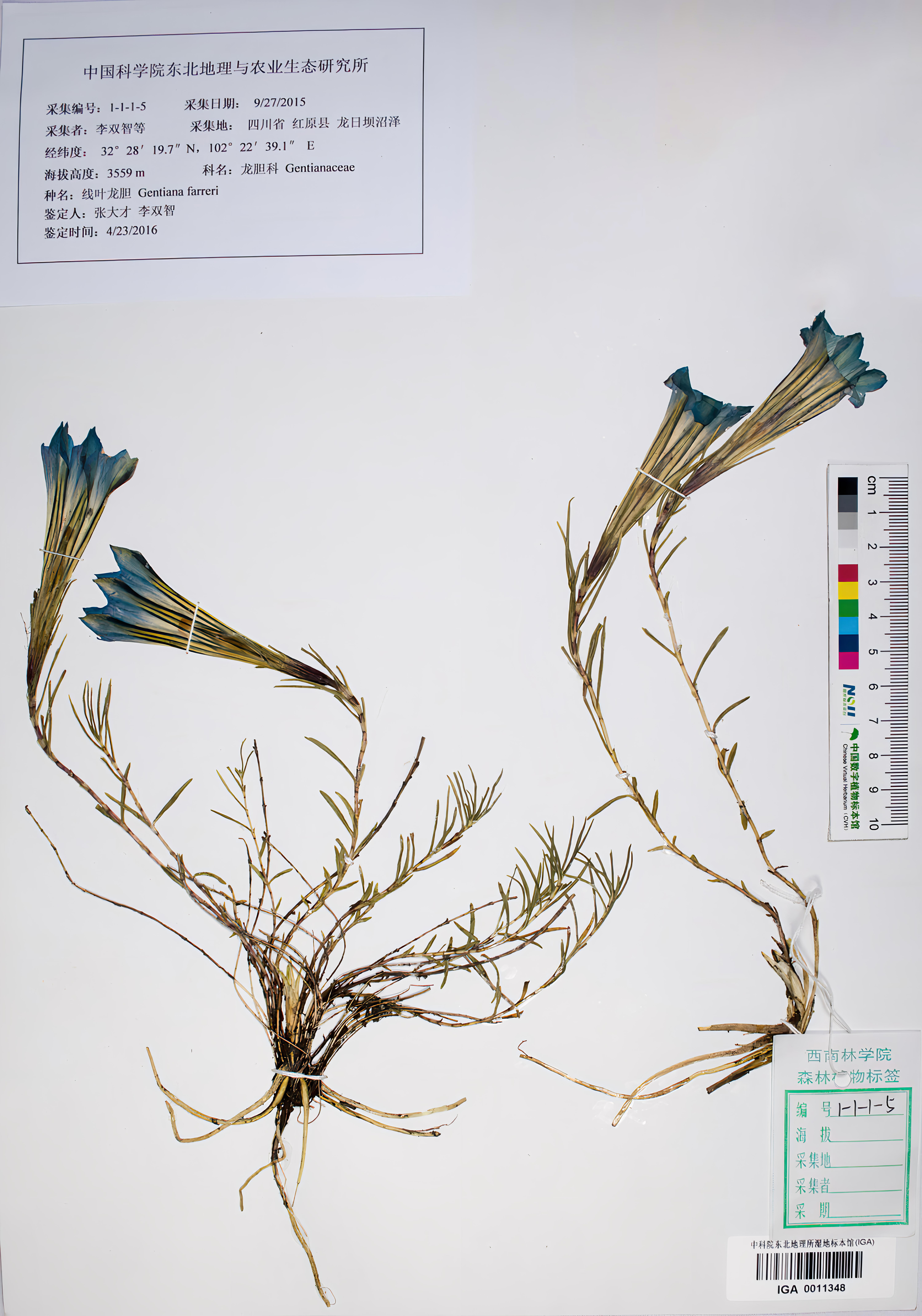

Supplement: Supplementary file 1 [file DataSheet1.ZIP › Herbarium pictures /G. lawrencei.jpg]

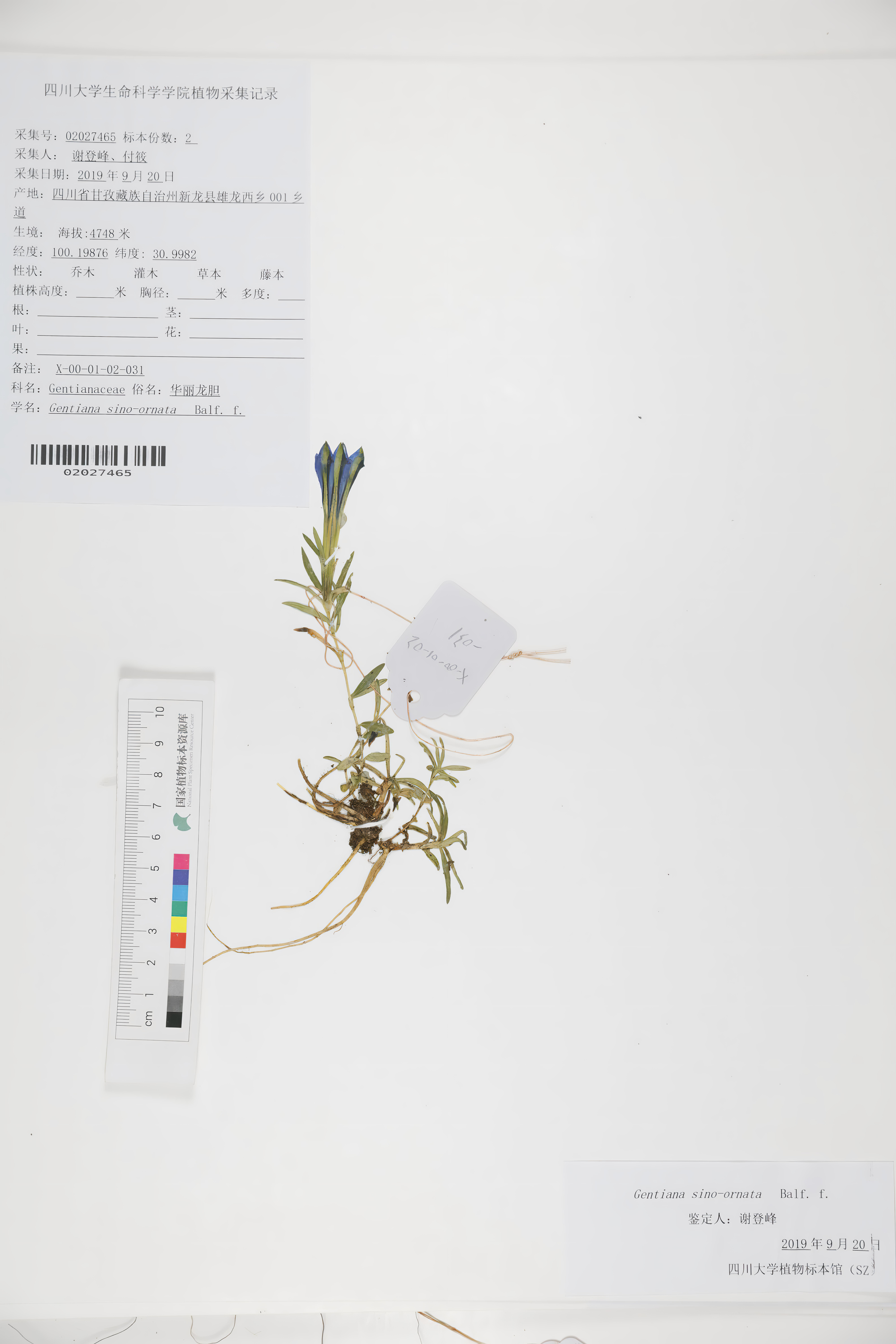

Supplement: Supplementary file 1 [file DataSheet1.ZIP › Herbarium pictures /G. sino-ornata.jpg]

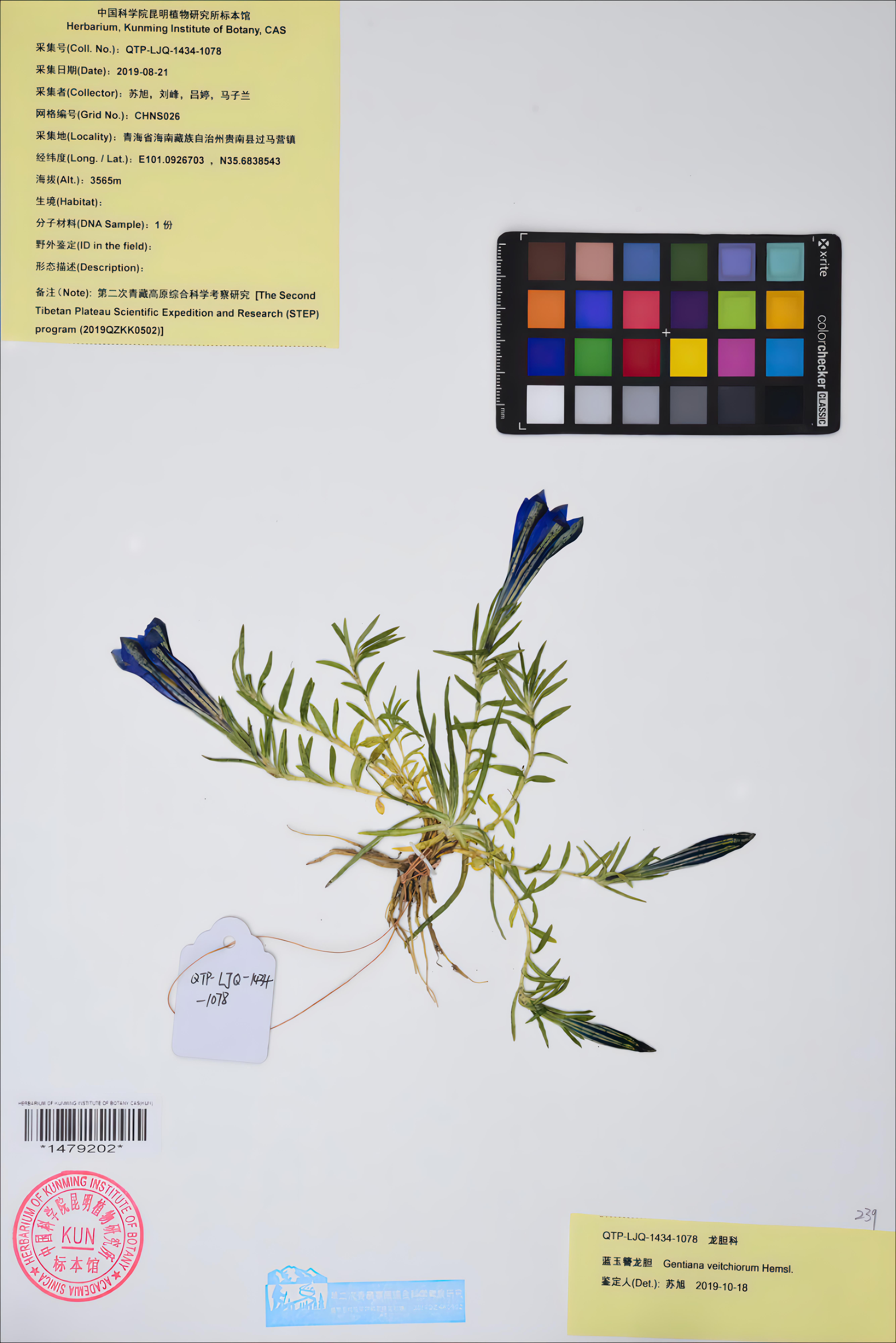

Supplement: Supplementary file 1 [file DataSheet1.ZIP › Herbarium pictures /G. veitchiorum.jpg]

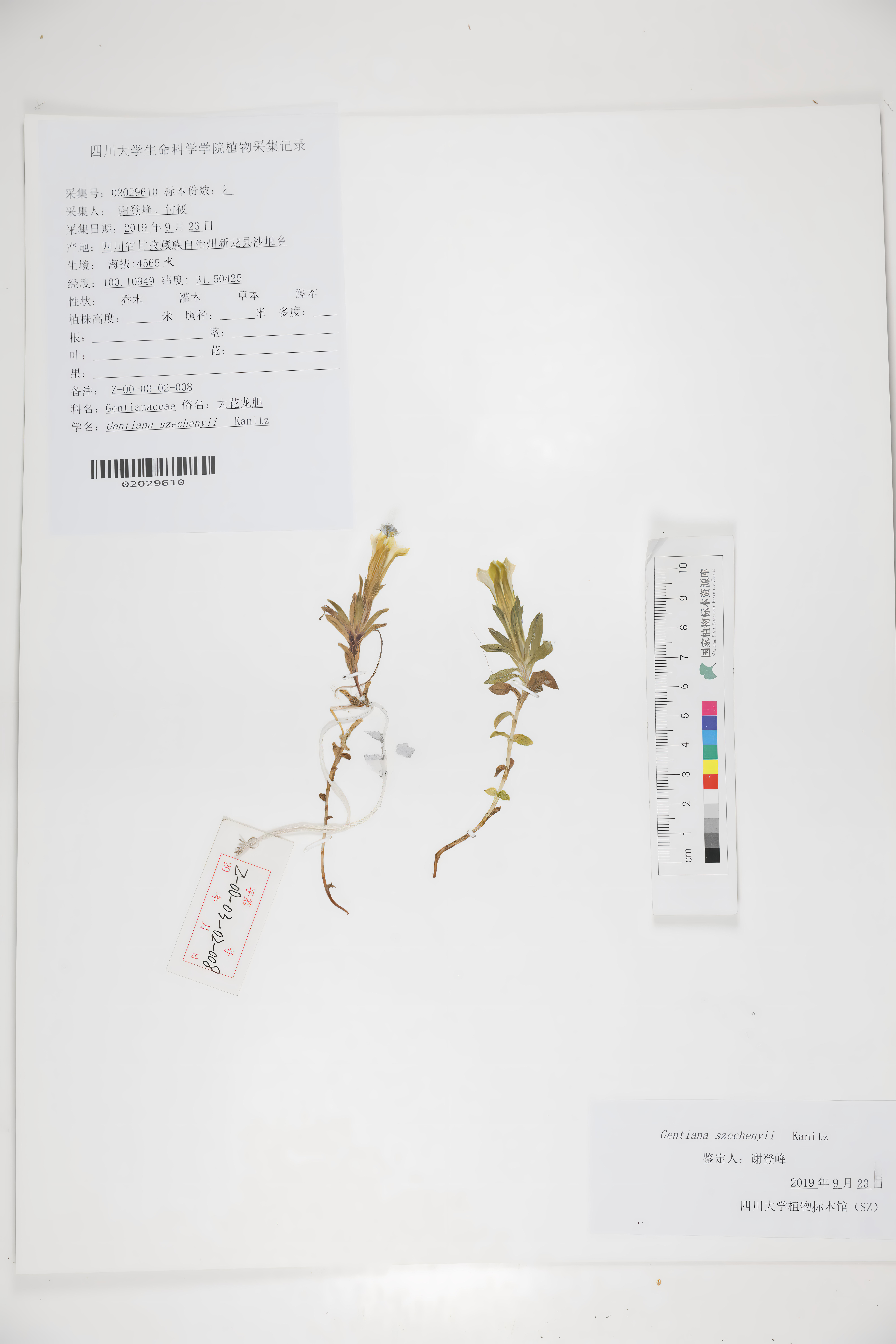

Supplement: Supplementary file 1 [file DataSheet1.ZIP › Herbarium pictures /G. szechenyii.jpeg]

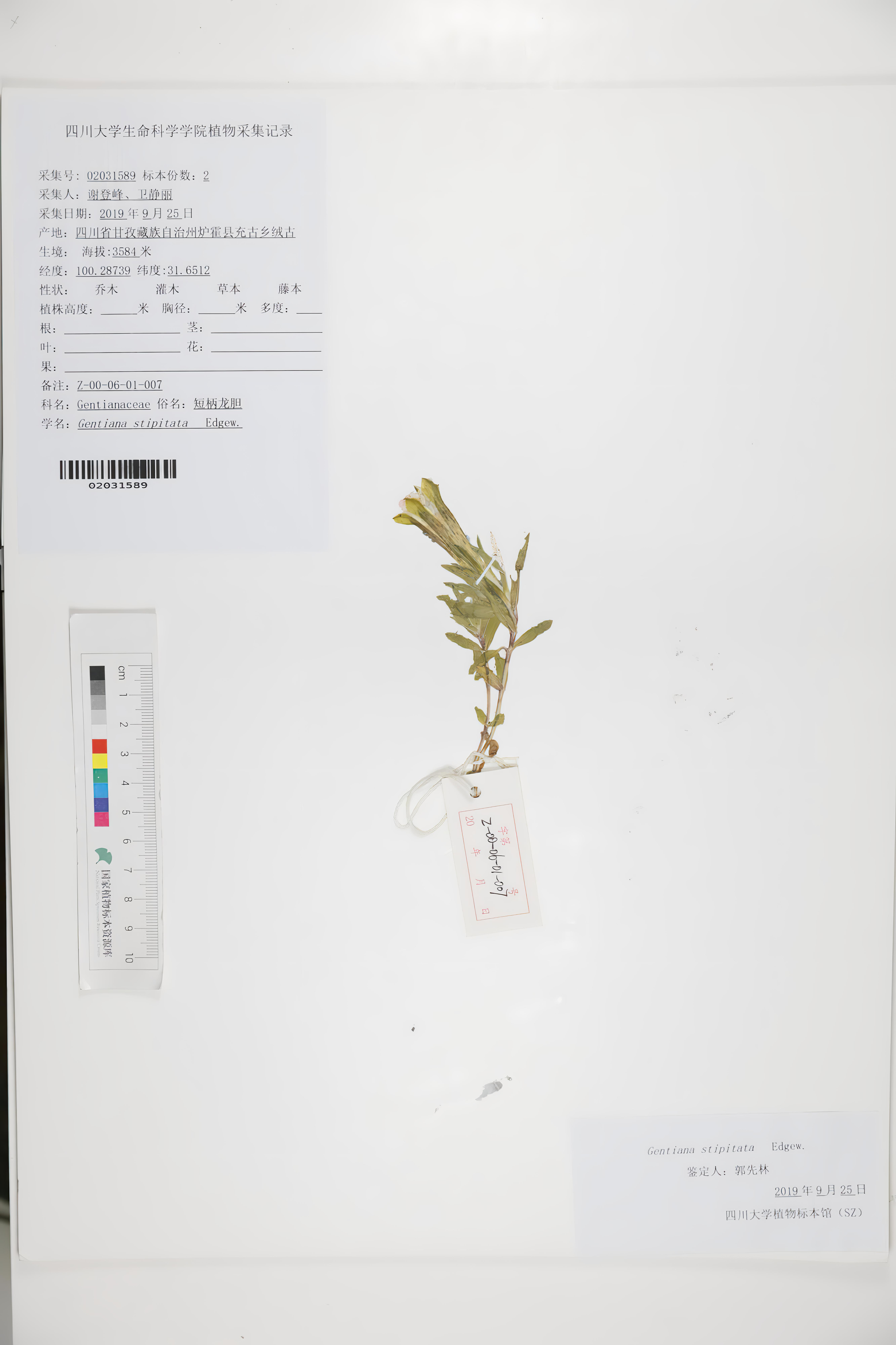

Supplement: Supplementary file 1 [file DataSheet1.ZIP › Herbarium pictures /G. stipitate.jpg]

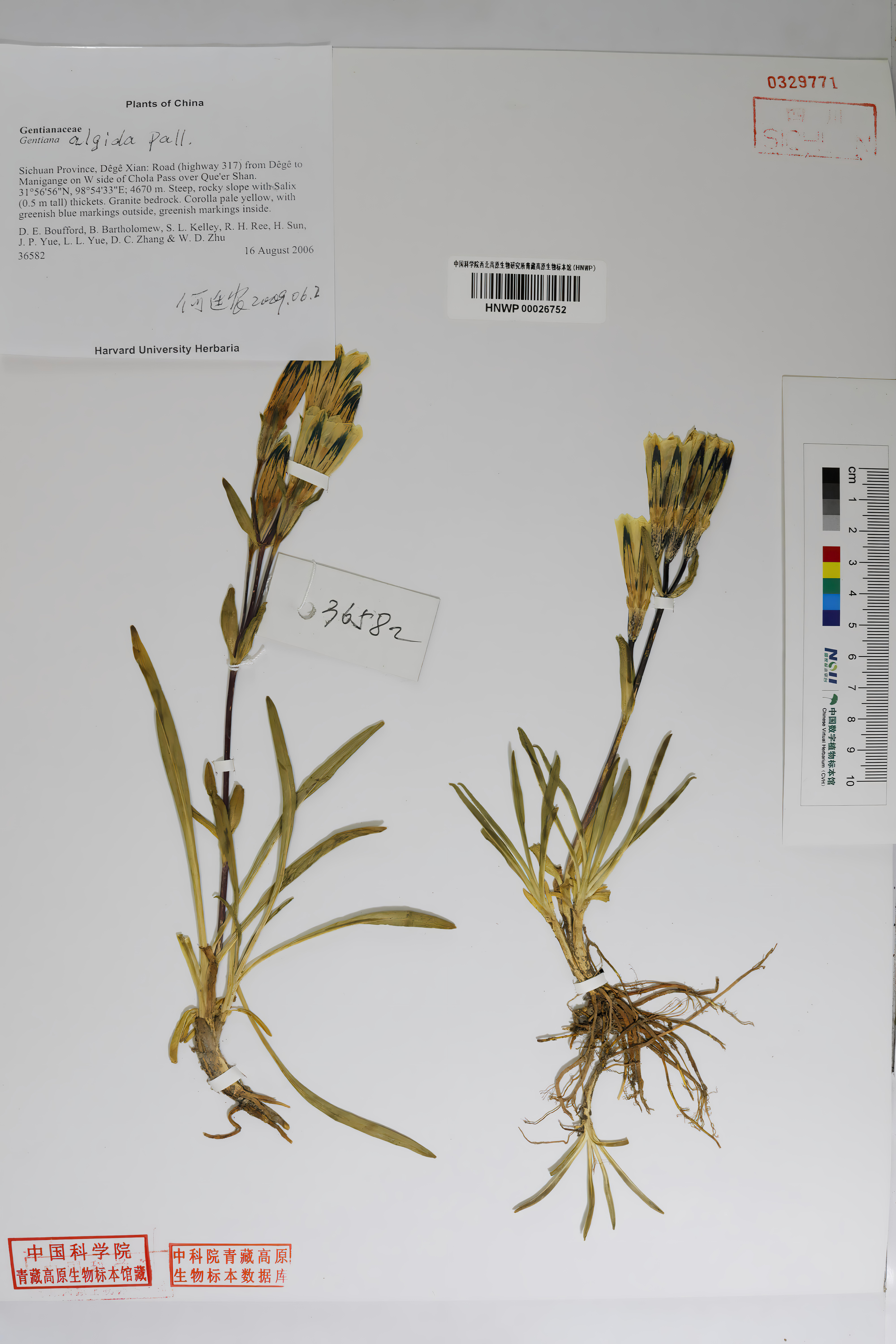

Supplement: Supplementary file 1 [file DataSheet1.ZIP › Herbarium pictures /G. algida.jpg]
